# Global incidence trends and projections of Alzheimer disease and other dementias: an age-period-cohort analysis 2021

**DOI:** 10.7189/jogh.15.04156

**Published:** 2025-05-23

**Authors:** Libo Xu, Zhenhao Wang, Mao Li, Qingsong Li

**Affiliations:** 1 The Second Affiliated Hospital of Harbin Medical University, Harbin, China; 2 University of California, Davis, California, USA

## Abstract

**Background:**

Alzheimer disease (AD) is a growing global health issue, with incidence varying by gender, age, and region. Understanding these trends is essential for developing effective prevention strategies as the population ages. Unlike previous Global Burden of Disease (GBD) studies that primarily focussed on prevalence and mortality, we offer a novel perspective by examining historical incidence trends and projecting future patterns of AD and other dementias using advanced analytical approaches.

**Methods:**

We used data from 204 countries and 21 global regions from the GBD 2021 database. We applied the age-period-cohort (APC) model to analyse historical incidence trends, and the Bayesian APC (BAPC) model to forecast future incidence from 2022–36. These models help reveal changes related to age, period, and birth cohort and enable forecasting of future trends – analytical perspectives not provided in the original GBD data sets or their supplementary documents.

**Results:**

Between 1992–2021, global AD cases increased from 4.078 million to 9.837 million, while the global age-standardised incidence rate (ASIR) remained relatively stable, rising slightly from 117.7 to 119.8 per 100 000. ASIR increased significantly in high-middle and middle-sociodemographic index regions, but declined in the low-sociodemographic index regions. Women consistently exhibited higher incidence rates than men across all regions. Projections indicate that 2036 global AD cases will reach 19.117 million, with an ASIR of 418.92 per 100 000.

**Conclusions:**

While global ASIR has remained stable, the number of AD cases continues to rise due to population ageing, particularly in middle- and high-income regions. Low-income regions face additional challenges due to limited health care resources. Gender disparities and unequal access to health care contribute to the variations in disease burden. These findings emphasise the need to prioritise early diagnosis and implement targeted interventions to reduce future disease burdens and address global health care inequalities.

Alzheimer disease (AD), a leading cause of dementia, along with other dementia types, ranks among the most prevalent neurodegenerative diseases worldwide. The Alzheimer Disease International predicts that the number of patients will double in the next 20 years, potentially triggering a major social crisis and prompting countries to implement comprehensive dementia prevention and control strategies [1]. According to the Global Burden of Disease (GBD) 2019 study, AD and other dementias, with AD being a primary form of dementia, are significant causes of death and disability [2,3]. Dementia, like many chronic diseases, severely impacts patients’ quality of life and imposes a substantial economic burden on families and society, though its progressive cognitive decline presents unique challenges [4]. The core pathological features of the disease include abnormal beta-amyloid deposition, pathological tau protein phosphorylation, and widespread loss of synapses and neurons [5,6]. These changes ultimately lead to a progressive decline in cognitive function, from mild memory impairment to severe cognitive loss and behavioural disturbances. Although significant advances have been made in understanding the disease's mechanisms, current treatments mainly target symptom relief, with no available cure [7].

The incidence of AD and other dementias, estimated globally at 9.8 million new cases (95% uncertainty interval (UI) = 8.6, 11.2 million) in 2021, is influenced by a variety of factors, including immutable genetic traits [8]. For instance, APOE ε4 gene variant carriers face a significantly increased risk [9,10]. However, environmental and lifestyle factors are critical in disease onset. Smoking, hypertension, diabetes, obesity, and low education levels have been identified as key risk factors for dementia [11,12].

Epidemiological data reveal significant differences in dementia incidence between developed and developing countries [13]. In high-income regions, such as North America, an estimated 7.0–8.0% of people aged ≥65 were living with AD and other dementias in 2019, with prevalence increasing sharply with age [11]. In contrast, lower-income and lower-middle-income countries, where medical resources are scarce and early diagnostic capabilities are limited, report lower dementia incidence rates [14]. Furthermore, global spending on AD treatment and care was estimated at USD 305 billion in 2020, with projections exceeding USD 1 trillion by 2050 [15], placing a considerable burden on society.

While prior GBD studies tracked the prevalence and mortality of AD and other dementias, incidence trends remain underexplored, limiting insights into new cases vital for early intervention as global ageing accelerates. We used age-period-cohort (APC) and Bayesian APC (BAPC) models to analyse incidence trends from 1992 to 2021 and predict rates to 2036, leveraging GBD 2021 data to inform public health strategies.

## METHODS

### Data sources and disease definitions

This study is based on data from the GBD 2021, which includes cross-sectional data collected between 1992 and 2021. The data set encompasses 21 global regions and 204 countries and territories, assessing the burden of 371 diseases and injuries, including AD and other dementias [8]. In the 10th revision of the International Classification of Diseases (ICD-10), AD is classified under code G30, which includes early-onset (G30.0), late-onset (G30.1), and other unspecified forms (G30.8 and G30.9). Dementia codes include AD with dementia (F00), vascular dementia (F01), dementia due to other diseases (F02), and unspecified dementia (F03). Data related to AD and other dementias were extracted from the GBD 2021 database, covering incident cases and incidence rates by gender (female, male, and total population), region (204 countries and territories), age (≥40 years), year (1992–2021), and the sociodemographic index (SDI). All incidence data used in this study were extracted from the GBD Results tool available on the VizHub platform developed by the Institute for Health Metrics and Evaluation [16]. Incidence rate refers to the number of new cases in a specific population during a given period. Using this data, our primary aim is to analyse historical incidence trends of AD and other dementias from 1992–2021 and predict future incidence rates up to 2036, with the age-standardised incidence rate (ASIR) per 100 000 persons as the primary outcome, accompanied by 95% UIs. Secondary outcomes include total incident cases and trends stratified by sex, age, and SDI regions. In the GBD tool, this indicator is expressed as the ratio of new cases to the mid-year population. The SDI ranges from zero (least developed) to one (most developed) and incorporates factors such as per capita gross domestic product (smoothed over the past 10 years), the average years of schooling for individuals aged ≥15, and the total fertility rate for people under 25 [17,18]. The University of Washington's institutional review board waived the need for informed consent to collect GBD data [19]. This study adheres to the Guidelines for Accurate and Transparent Health Estimates Reporting [20].

GBD data sources are subject to rigorous screening, integrating information from government and international websites, peer-reviewed reports, and other authoritative sources. These data cover various health information, including census data, household surveys, vital records, and disease registries. All data are categorised and indexed in the Global Health Data Exchange. The burden of AD and other dementias is modeled using the Bayesian meta-regression tool DisMod-MR 2.1, which adjusts for biases in non-reference data and accounts for incomplete or missing data across regions to estimate AD incidence, including underdiagnosis in low-resource settings and limitations of historical data up to 2021, by correcting incomplete reporting based on regional health care access and data quality [8]. We relied on these pre-corrected GBD 2021 estimates as a secondary analysis with no additional bias adjustments applied in the APC and BAPC analyses.

### Overall time trend analysis of AD and other dementias incidence

To estimate the ASIR of AD and other dementias, we used a direct method for age standardisation, assuming that the incidence rate is the weighted sum of independent Poisson random variables.

### APC model analysis

We employed the APC model to analyse the impact of age, period, and cohort effects on the incidence of AD and other dementias [21]. APC analysis is used to explore disease trends by considering age effects, period effects (population-level changes), and cohort effects (early-life or generational exposures) [22,23]. Covariates including age, sex, and SDI were selected based on their established associations with AD and other dementia incidence, as evidenced by prior GBD studies and epidemiological literature, which highlight age as the primary risk factor, sex differences in disease susceptibility, and SDI as a proxy for socioeconomic and health care access variations [8,11]. These covariates enable a comprehensive assessment of incidence trends across diverse populations and time periods. Net drift represents the overall annual percentage change. In contrast, local drift reflects the annual percentage change for each age group [24]. The longitudinal age curve illustrates the fitted age-specific incidence rates for the reference cohort after adjusting for period deviations. Period relative risks (RRs) measure the incidence risk in each period relative to the reference period, adjusted for age and nonlinear cohort effects. Cohort RRs indicate the incidence risk in each birth cohort relative to the reference cohort, adjusted for age and nonlinear period effects. An RR greater than 1 signifies an increased incidence risk, while an RR<1 indicates a decreased risk.

To address the identification problem in APC analysis, we applied the intrinsic estimator (IE) method using the APC web tool from the USA National Cancer Institute [25]. The IE method ensures unique parameter estimates by applying an orthogonal constraint that resolves collinearity among age, period, and cohort effects, focussing on estimable functions like net drift and local drift to produce robust AD incidence trends [18]. We selected IE over alternatives such as constrained generalised linear models due to its widespread acceptance in epidemiology and ability to provide stable estimates without subjective constraints.

### Prediction

Using the BAPC model with the integrated nested Laplaces approximation method, we predicted AD and other dementia incidence rates from 2022–36 [26]. The BAPC model employed default priors and hyperparameters within the integrated nested Laplace approximation framework, as provided by the *R* package BAPC, which are informed by broad epidemiological trends and validated against GBD 2021 historical data, ensuring robust and stable forecasts [8,26].

### Statistical analysis

We expressed ASIR estimates per 100 000 people, along with 95% UI. We performed data analyses and visualisations using *R*, version 4.4.0 (R Core Team, Vienna, Austria), with packages including BAPC (version 0.0.36), INLA (version 24.05.10), and ggplot2 (version 3.5.1). We determined statistical significance by a two-sided *P*-value of <0.05. We used a 95% confidence level for UIs, calculated as Bayesian credible intervals from BAPC and DisMod-MR 2.1 models, consistent with GBD standards [8]. We chose BAPC because of its predictive accuracy, and the Poisson distribution was assumed for ASIR due to its fit for count data.

## RESULTS

### Trends in global and SDI region incidence rates of AD and other dementias

**Figure 1:**
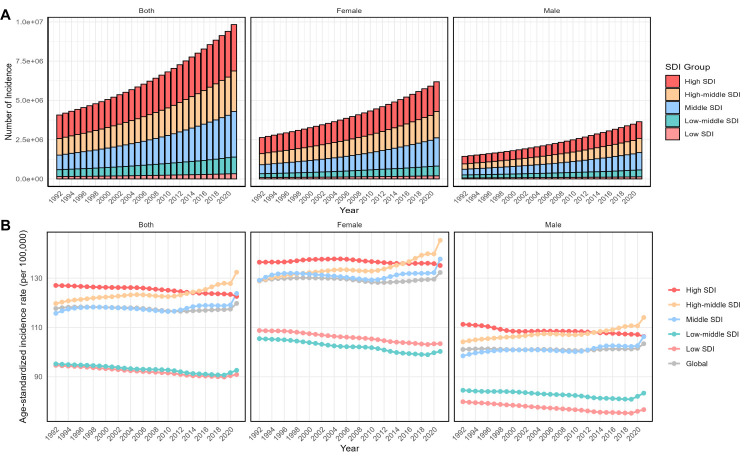
Global and five SDI regions’ incidence and ASIR of AD and other dementias (1992–2021). **Panel A.** Number of incident cases. **Panel B.** ASIR (per 100 000 persons). AD – Alzheimer disease, ASIR – age-standardised incidence rate.

Globally, AD cases rose from 4.078 million (95% UI = 3.587, 4.630 million) in 1992 to 9.837 million (95% UI = 8.621, 11.164 million) in 2021 (**Figure 1**, Panels A and B; **Table 1**). Despite the increase in global cases, the ASIR remained nearly constant during the same period, increasing only slightly from 117.7 cases per 100 000 (95% UI = 103.7, 133.0) in 1992 to 119.8 cases per 100 000 (95% UI = 105.0, 135.9) in 2021, with APC of 0.013% (95% confidence interval (CI) = −0.002, 0.029).

**Table1:** Global and SDI incidence trends of AD and other dementias (1992–2021)

	1992		2021		
	**Number (95% UI)**	**ASIR (95% UI)**	**Number (95% UI)**	**ASIR (95% UI)**	**APC net drift (95% CI)**
**Global**	4 077 542 (3 587 065, 4 630 267)	117.69 (103.69, 133.01)	9 837 056 (8 620 519, 11 163 700)	119.76 (104.96, 135.89)	0.013 (−0.002, 0.029)
**High SDI**	1 500 526 (1 326 297, 1 690 270)	127.05 (112.62, 142.25)	2 952 147 (2 586 722, 3 344 548)	122.61 (107.45, 138.44)	−0.002 (−0.037, 0.034)
**High-middle SDI**	1 054 854 (919 298, 1 211 423)	119.71 (104.93, 135.89)	2 582 346 (2 252 029, 2 941 774)	132.4 (115.43, 150.85)	0.21 (0.176, 0.244)
**Middle SDI**	926 754 (809 182, 1 056 770)	115.73 (101.29, 131.64)	2 902 080 (2 542 320, 3 314 767)	123.79 (108.25, 141.26)	0.058 (0.035, 0.081)
**Low-middle SDI**	439 242 (384 345, 498 412)	95.21 (83.01, 108.34)	1 063 277 (929 612, 1 207 790)	92.61 (80.79, 105.71)	−0.134 (−0.147, −0.12)
**Low SDI**	151 469 (132 165, 171 831)	94.64 (82.4, 107.72)	328 703 (287 112, 372 981)	90.89 (79, 103.12)	−0.188 (−0.212, −0.164)

APC – age-period-cohort, ASIR – age-standardised incidence rate, CI – confidence interval, SDI – sociodemographic index, UI – uncertainty interval

Significant differences in incidence and ASIR trends were observed across different SDI regions. In high-middle SDI regions, the number of cases rose from 1.055 million (95% UI = 0.919, 1.211 million) in 1992 to 2.582 million (95% UI = 2.252, 2.942 million) in 2021, and the ASIR rose from 119.7 cases per 100 000 (95% UI = 104.9, 135.9) to 132.4 cases per 100 000 (95% UI = 115.4, 150.9), with an APC of 0.21% (95% CI = 0.176, 0.244), indicating a significant rise in incidence. In middle SDI regions, ASIR increased from 115.7 cases per 100 000 (95% UI = 101.3, 131.6) in 1992 to 123.8 cases per 100 000 (95% UI = 108.3, 141.3) in 2021, with an APC of 0.058% (95% CI = 0.035, 0.081). Conversely, ASIR declined in low and low-middle SDI regions. In low SDI regions, ASIR decreased from 94.6 cases per 100 000 (95% UI = 82.4, 107.7) in 1992 to 90.9 cases per 100 000 (95% UI = 79.0, 103.1) in 2021, with an APC of −0.188% (95% CI = −0.212, −0.164). Similarly, in low-middle SDI regions, ASIR declined from 95.2 cases per 100 000 (95% UI = 83.0, 108.3) to 92.6 cases per 100 000 (95% UI = 80.8, 105.7), with an APC of −0.134% (95% CI = −0.147, −0.12). In all regions, both incidence and ASIR were significantly higher in females than in males.

Overall, despite the increase in global case numbers, ASIR tends to decline in high SDI regions, while incidence and ASIR continue to rise in upper-middle and middle-income countries, reflecting growing global health inequalities.

### National trends in incidence rates of AD and other dementias

From 1992 to 2021, the incidence of AD and other dementias continued to rise in many countries, particularly in upper-middle and middle SDI regions (Figure S1, Panels A and B, and Table S1 in the **Online Supplementary Document**). In 2021, China (n = 2.914 million; 95% UI = 2.504, 3.351 million) and India (n = 0.749 million; 95% UI = 0.649, 0.857 million) recorded the highest number of AD and other dementia cases, accounting for more than 30% of the global total. In contrast, Niue and Nauru reported the fewest cases. In 2021, the countries with the highest ASIRs per 100 000 were Lebanon (n = 140.48; 95% UI = 122.53, 159.62) and Iraq (n = 129.62; 95% UI = 113.05, 148.98), while India (n = 78.92; 95% UI = 68.29, 90.58) and Bangladesh (n = 79.47 cases; 95% UI = 68.72, 90.87) had the lowest ASIRs per 100 000. Taiwan exhibited the largest increase in ASIR (0.48%; 95% UI = 0.38, 0.57), while Denmark experienced the largest decline (−0.72%; 95% UI = −0.88, −0.57).

### Time trends of AD and other dementias in different age groups

In 1992, approximately 25% of cases occurred in individuals >65 years (Figure S2, Panel A in the **Online Supplementary Document**). Between 1992 and 2021, the incidence of AD and other dementias steadily rose in individuals aged ≥65. Similar trends were observed across all five SDI regions.

Globally, local drift values for individuals aged 50–74 were above zero, indicating increased incidence in these groups (Figure S2, Panel B in the **Online Supplementary Document**). This trend was similarly observed in high, upper-middle, and middle SDI regions. Conversely, in low and low-middle SDI regions, local drift values for all age groups were below zero from 1992 to 2021, reflecting a decline in AD and dementia incidence across all age groups.

Notably, gender also influenced changes in incidence rates. In all SDI regions, the annual percentage change in incidence was generally higher for older females than males, particularly in high SDI regions, where the increase in female incidence was more pronounced. This may be attributed to women's longer life expectancy, increasing their vulnerability to AD and other dementias in later life.

### Influence of age, period, and cohort on the incidence of AD and other dementias

**Figure 2:**
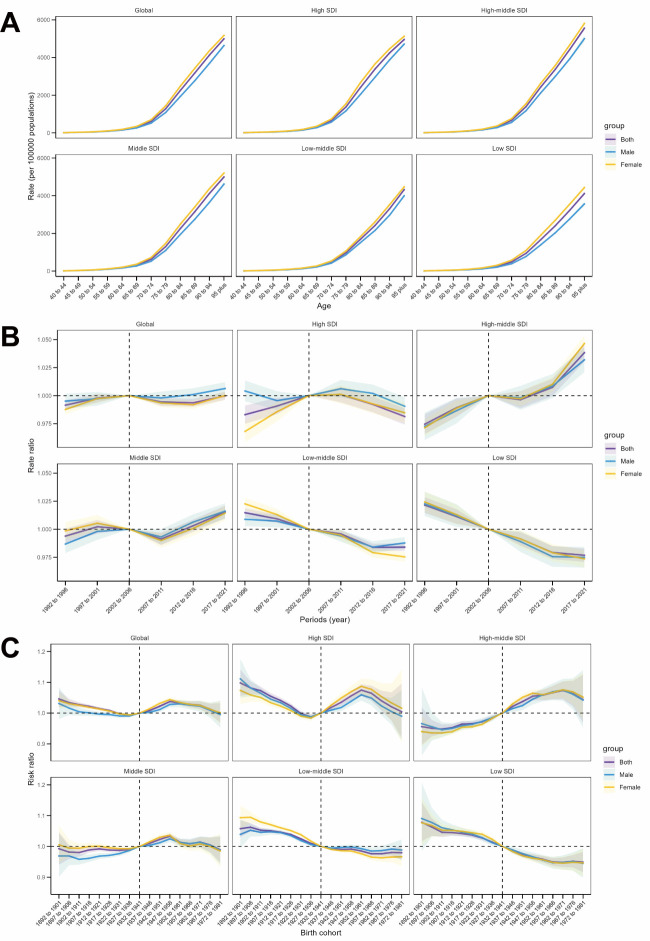
Age, period, and birth cohort effects on the incidence of AD and other dementias analysed using APC models.* **Panel A.** Age effect. **Panel B.** Period effects. **Panel C.** Birth cohort effects. AD – Alzheimer disease, APC – age-period-cohort. *The shaded areas depict incidence rates or rate ratios with their respective 95% confidence intervals.

Overall, age effects were consistent across SDI regions, with the risk of AD and other dementias increasing with age and peaking in individuals aged ≥95 (**Figure 2**, Panels A–C). The incidence risk was also higher for females than males in all SDI regions (**Figure 2**, Panel A).

Period effects indicated stable incidence rates without significant increases (**Figure 2**, Panel B). In high SDI regions, period effects initially exhibited a slight upward trend, followed by a decline in incidence rates after 2012 (**Figure 2**, Panel B). A similar upward trend was observed in upper-middle and middle SDI regions, where incidence rates continued to rise. During the study period, upper-middle and middle SDI regions faced unfavourable period risks, while low and low-middle SDI regions exhibited favourable period risks, with overall declining trends. Additionally, gender differences in period risks were observed.

Regarding cohort effects, globally, the incidence risk followed a pattern of initial decline, followed by an increase, and eventually a slight decrease across successive birth cohorts, resulting in an overall mild downward trend. Trends in high and middle SDI regions largely followed the global pattern. Notably, cohort analyses in low-middle and low SDI regions revealed gradual improvements in incidence risk across successive birth cohorts (**Figure 2**, Panel C).

To better illustrate the temporal trends in the global incidence of AD and other dementias, we presented typical countries for several age, period, and birth cohort effects (Figure S3 in the **Online Supplementary Document**).

### Forecasting AD and other dementias from 2022–36

**Figure 3:**
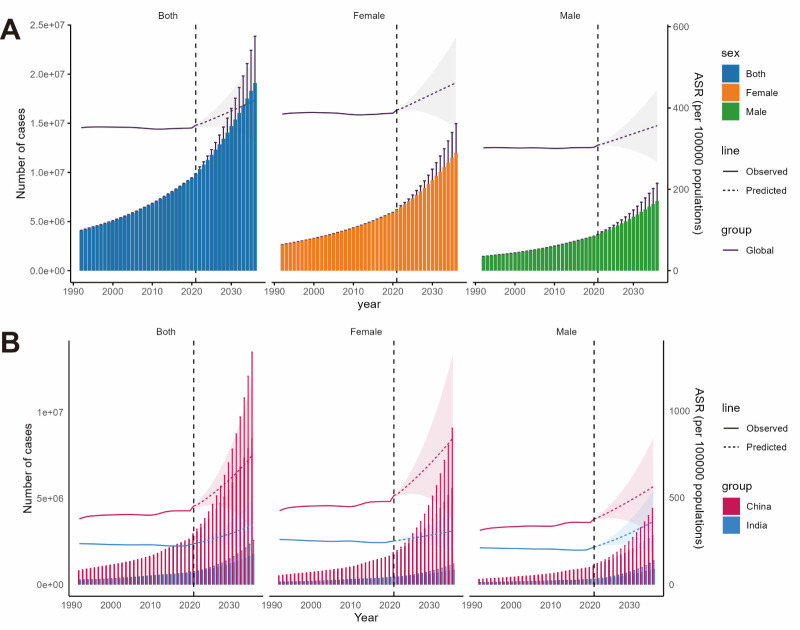
The ASIR and the number of cases of AD and other dementias from 2022–36. **Panel A.** Global. **Panel B.** China and India. AD – Alzheimer disease, ASIR – age-standardised incidence rate.

The BAPC model predicted future trends in ASIR and case numbers for AD and other dementias from 2022–36. The number of AD and other dementia cases worldwide is expected to rise, reaching 19.117 million (95% CI = 14.381, 23.854 million) by 2036 (**Figure 3**, Panel A; Table S2 in the **Online Supplementary Document**). Meanwhile, ASIR is projected to increase significantly, reaching 418.92 cases per 100 000 people (95% CI = 315.03, 522.8) by 2036, although this remains lower than historical peaks.

The model predicts that the number of cases and ASIR will continue to rise in China and India (**Figure 3**, Panel B). Although their ASIR trends are similar to global trends, the situation in China is more severe, with a higher ASIR than in India. Furthermore, the ASIR in Chinese females is expected to increase faster than in males after 2022, while the rise in ASIR among Indian females is projected to be slower than that of males.

## DISCUSSION

This study, through an analysis of global incidence trends of AD and other dementias across various SDI regions, reveals that despite the relatively stable global ASIR over the past three decades, the sharp rise in case numbers, especially in upper-middle and middle SDI countries, reflects worsening global health inequalities. Our findings show that while the incidence rate stabilises in high-income countries, incidence and case numbers continue to increase in middle-income countries. This trend further underscores the impact of global population ageing and unequal resource distribution on disease burden.

Although the global ASIR for AD and other dementias has remained relatively stable, the increase in total cases nearly mirrors the global ageing process. In high-income countries, disease burden management is relatively well-developed, and early interventions and treatments help slow disease progression. In contrast, low- and middle-income countries (LMICs) face challenges related to resource scarcity, including insufficient public health infrastructure, preventive measures, and diagnostic equipment [27]. This imbalance accounts for the significant increase in global case numbers despite relatively stable ASIR. In high-income countries, increased resource investment has improved early screening, diagnosis, and treatment of dementia, slowing the upward trend in ASIR [28,29]. Specifically, in high-SDI regions, improved diagnostic practices likely enhance early detection, contributing to apparent stabilisation rather than a true reduction in incidence, whereas in the middle-SDI regions, rising rates may reflect improved reporting and increasing underlying incidence due to limited interventions.

From 2020–21, a sudden increase in the ASIR was observed in most SDI regions, which may be closely related to the impact of the COVID-19 pandemic on global health care systems and the elderly population. First, the COVID-19 pandemic led to the widespread redirection of health care resources to emergency care and pandemic control, severely disrupting routine medical services such as chronic disease management and early diagnosis of dementia [30]. Many elderly patients in various countries failed to receive timely diagnosis and treatment, worsening their conditions and contributing to a short-term increase in AD. Second, the social isolation brought about by the pandemic had profound adverse effects on elderly individuals, particularly those with pre-existing cognitive impairments. Long-term isolation, lack of social support, and psychological stress may have accelerated cognitive decline, resulting in a rise in AD and other dementia cases [31,32]. Additionally, the direct impact of COVID-19 on the nervous system, particularly the neurocognitive damage associated with long COVID symptoms, may have further contributed to the increase in incidence during this period [33]. Given the pandemic’s disruption to health care systems noted in our analysis, this spike likely reflects a short-term increase, potentially accentuated by reporting challenges. However, GBD 2021 does not explicitly flag such uncertainty.

This study further confirms the significant role of gender in AD, particularly in high-income countries, where incidence rates are notably higher among women than men. This gender disparity may partly result from biological mechanisms, including the loss of estrogen's neuroprotective effects in postmenopausal women, which accelerates the progression of neurodegenerative diseases [34,35]. Estrogen reduction may increase the accumulation of beta-amyloid and the phosphorylation of tau proteins, both hallmark pathological features of AD [36]. Moreover, women generally live longer than men, making them more likely to reach the high-risk age group for AD, further contributing to the higher incidence among women. This gender disparity is evident not only in high-income countries but also in LMICs, where women may face greater socioeconomic challenges and fewer opportunities for early intervention and medical support. In low-SDI regions, the gender gap appears less pronounced than in the high-SDI regions, likely because unequal access to scarce medical resources disadvantages women more than men, potentially underrepresenting female incidence due to lower diagnosis rates influenced by social norms prioritising male health care.

China and India, the two most populous countries globally, also account for the highest number of AD and dementia cases. These countries face distinct challenges in managing AD due to their asynchronous ageing processes and uneven health care resource distribution. China's rising ASIR is particularly concerning, likely tied to its rapidly ageing population and urbanisation. Urbanisation may erode traditional family care models, leaving the elderly without adequate support during the early stages of the disease. By contrast, India's relatively lower ASIR can be attributed to its younger population and lower proportion of elderly individuals in rural areas. However, as India's population ages, the country will likely face a more severe AD burden in the future. Additionally, the high ASIR in Middle Eastern countries like Lebanon and Iraq indicates the complex interplay of social unrest, cultural factors, and health care system challenges contributing to the dementia burden [37]. Similarly, in high-SDI regions like Taiwan and Denmark, which show the largest increase (net drift = 0.48%) and decrease (net drift = −0.72%) in ASIR respectively, abundant health care resources may heighten sensitivity to mild disease changes, potentially contributing to Taiwan’s rise through increased detection amid rapid ageing and to Denmark’s decline through effective early interventions.

Age effects are consistent across all SDI regions, with AD risk rising sharply with age and peaking at 95 years and older. This trend reflects AD primary pathological mechanisms, where the abnormal accumulation of beta-amyloid and tau proteins accelerates with age, causing irreversible neuronal damage [38,39]. Moreover, period effects highlight variations in disease screening and management across countries and regions. High-income countries benefit from early diagnosis and effective management, resulting in declining or stable incidence rates, while LMICs, constrained by limited resources, bear a significant disease burden [40].

AD incidence is influenced by both biological and socioeconomic factors, as well as environmental conditions. Lifestyle-related risk factors such as cardiovascular disease, hypertension, diabetes, and obesity are increasing in middle-income countries, and these factors are closely linked to AD [41,42]. The rapid pace of urbanisation, lifestyle changes, and the breakdown of traditional family structures in LMICs may exacerbate the accumulation of these risk factors. Furthermore, lower levels of education and cognitive reserve make populations in these regions more vulnerable to AD, emphasising the need for better education and preventive measures [43].

Globally, the number of AD and dementia cases is expected to rise, particularly in LMICs. These countries may face significant public health challenges in addressing rapid ageing and the growing dementia burden. As the ageing process accelerates in populous countries such as China and India, public health strategies must focus on gender differences and health management for elderly women. Moreover, the lingering impact of the COVID-19 pandemic on chronic disease management indicates that future public health policies must better balance emergency medical services with chronic disease management. This BAPC forecast represents a status quo scenario based on current trends, and future breakthroughs in disease-modifying therapies could potentially alter these projections significantly.

The complex pathological mechanisms of AD, including the accumulation of beta-amyloid, tau proteins, neuroinflammation, and oxidative stress, manifest differently across socioeconomic contexts. Future research should explore how these biological mechanisms interact with social and environmental factors to accelerate or delay disease progression. Molecular studies focussing on different genders, regions, and populations can aid in developing more targeted treatment and prevention strategies. Globally, addressing the AD burden still requires more efforts in resource allocation, early diagnosis, and personalised treatment.

Although this study reveals trends in AD incidence globally and across various SDI regions, several limitations exist. First, as the data primarily rely on the GBD database, disease reporting and data collection in some LMICs may be incomplete, potentially underestimating incidence rates in these regions. The DisMod-MR 2.1 tool mitigates this underreporting through Bayesian meta-regression, adjusting for incomplete data using regional health care access and multiple data sources. However, misclassifying AD and other dementias may still occur due to diagnostic variability across regions, and residual missing data could affect trend accuracy. No additional data imputation was performed in this secondary analysis, relying instead on GBD 2021’s pre-corrected estimates. Additionally, the model's predictions are based on existing data and do not fully account for future socioeconomic changes, health care resource distribution, or intervention improvements. Second, while the GBD database provides comprehensive demographic analysis, it does not deeply explore how cultural differences, social support systems, and lifestyle factors interact with disease pathogenesis, potentially limiting insights into incidence variations, particularly in LMICs. Finally, due to the transnational and long-term nature of the data, inconsistencies or quality differences may exist across countries, which could influence model accuracy and result reliability. Therefore, future research should focus on improving AD data quality in LMICs and further investigate the relationship between environmental factors and disease progression.

## CONCLUSIONS

This study highlights significant global trends in the incidence of AD and other dementias, alongside their complex relationship with global health inequalities. While global ASIR remains relatively stable, the growing burden of the disease, particularly in middle-income countries, is driven by population ageing and worsening socioeconomic inequalities. Gender differences play a critical role in AD incidence, and future public health policies should prioritise managing the health of elderly women. To address this escalating global health challenge, targeted intervention strategies are necessary to reduce the burden of AD and enhance the quality of life for affected populations. To enable timely interventions, practitioners should prioritise early screening programs in ageing populations, particularly in high-SDI regions with rising incidence. Future research should refine incidence predictions by incorporating emerging risk factors like air pollution and assessing the impact of new diagnostic tools on early detection rates. Policymakers should focus on increasing funding for dementia care infrastructure in LMICs to address projected incidence increases.

## Additional material


Online Supplementary Document
