# Supplementary material for: Global incidence trends and projections of Alzheimer disease and other dementias: an age-period-cohort analysis 2021
Source: J Glob Health. 2025 May 23;15:04156. doi: 10.7189/jogh.15.04156 (PMC12100573; doi:10.7189/jogh.15.04156)

Table S1. Changes in national incidence rates of AD and other dementias from 1992 to 2021.

location	1992		2021		APC
	Number (95%UI)	ASIR (95%UI)	Number (95%UI)	ASIR (95%UI)	Netdrift (95%CI)
Lao People's Democratic Republic	1660 (1430 to 1896)	113.69 (99.88 to 129.87)	3764 (3280 to 4290)	111.82 (97.43 to 127.89)	-0.03 (-0.26 to 0.21)
Timor-Leste	223 (195 to 251)	119.3 (103.86 to 136.18)	768 (666 to 888)	113.2 (98.46 to 129.74)	-0.18 (-0.72 to 0.36)
Georgia	6594 (5771 to 7525)	113.79 (99.43 to 130.01)	7576 (6603 to 8611)	113.98 (99.53 to 129.49)	0.03 (-0.14 to 0.19)
Solomon Islands	97 (83 to 111)	112.84 (98.27 to 129.48)	256 (219 to 295)	113.74 (98.07 to 131.08)	0.24 (-1.04 to 1.53)
Democratic People's Republic of Korea	13116 (11272 to 15096)	110.97 (95.88 to 127.95)	31408 (26865 to 36668)	108.5 (93.89 to 125.37)	-0.12 (-0.2 to -0.03)
Maldives	68 (58 to 77)	109.88 (95.92 to 125.48)	309 (269 to 355)	113.22 (98.24 to 129.67)	0.25 (-1.05 to 1.56)
Bosnia and Herzegovina	3735 (3220 to 4293)	111.74 (97.28 to 128.13)	7140 (6148 to 8275)	111.79 (96.79 to 127.27)	0.02 (-0.15 to 0.2)
Vanuatu	44 (37 to 51)	107.72 (93.33 to 123.98)	119 (101 to 138)	105.3 (90.64 to 121.61)	0.1 (-1.47 to 1.7)
Kyrgyzstan	2955 (2576 to 3359)	112.64 (98.22 to 128.38)	4134 (3622 to 4676)	113.88 (99.39 to 130.07)	0.09 (-0.11 to 0.29)
Kiribati	29 (25 to 33)	121.44 (105.53 to 138.95)	54 (46 to 62)	121.84 (106.12 to 138.96)	-0.1 (-2.06 to 1.89)
Philippines	27813 (24209 to 31882)	118.99 (104.75 to 135.44)	72092 (63052 to 81980)	113.91 (99.74 to 130)	-0.21 (-0.27 to -0.15)
Croatia	6148 (5344 to 7085)	115.77 (101.1 to 133.22)	11299 (9711 to 12993)	112.67 (98.23 to 128.39)	-0.04 (-0.19 to 0.11)

Tajikistan	2626 (2296 to 2978)	111.49 (97.32 to 126.93)	4157 (3626 to 4715)	104.98 (92.03 to 120.4)	-0.18 (-0.38 to 0.02)
Thailand	29905 (26103 to 33962)	101.98 (88.82 to 115.8)	112595 (98738 to 127875)	103.27 (90.64 to 117.58)	0.13 (0.08 to 0.18)
Micronesia (Federated States of)	43 (37 to 51)	119.51 (103.39 to 137)	56 (48 to 64)	120.71 (104.84 to 139.46)	0.34 (-1.43 to 2.13)
Indonesia	84100 (73439 to 95482)	115.95 (101.29 to 132.28)	189722 (164142 to 216604)	113.31 (98.38 to 129.52)	-0.09 (-0.12 to -0.06)
Uzbekistan	11398 (9888 to 13130)	106.85 (93 to 122.73)	20088 (17467 to 22885)	106.19 (92.22 to 122.08)	0 (-0.1 to 0.09)
Samoa	73 (62 to 84)	111.57 (96.41 to 128.78)	125 (107 to 144)	108.37 (93.13 to 125.19)	-0.19 (-1.33 to 0.95)
Azerbaijan	4820 (4212 to 5530)	113.7 (99.47 to 130.16)	8311 (7218 to 9441)	110.27 (96.65 to 126.18)	-0.11 (-0.25 to 0.04)
Albania	1929 (1688 to 2194)	111.73 (97.26 to 127.96)	4828 (4174 to 5529)	112.62 (98 to 128.7)	0.06 (-0.16 to 0.28)
China	797869 (685680 to 915875)	126.98 (110.73 to 144.66)	2914112 (2504728 to 3350743)	151.47 (131.22 to 173.34)	0.35 (0.31 to 0.4)
Malaysia	9527 (8380 to 10775)	116.29 (101.79 to 132.63)	26046 (22443 to 29858)	111.29 (95.99 to 127.46)	-0.12 (-0.2 to -0.03)
Poland	49371 (42659 to 56817)	120.12 (104.53 to 137)	88407 (76685 to 101187)	114.27 (99.44 to 130.4)	-0.17 (-0.22 to -0.12)
Iceland	429 (375 to 485)	134.35 (117.61 to 151.24)	799 (689 to 898)	122.36 (106.34 to 137.53)	-0.37 (-1.02 to 0.28)
Denmark	9424 (8279 to 10766)	102.16 (90.11 to 115.97)	11466 (9906 to 13148)	84.34 (72.78 to 96.21)	-0.72 (-0.88 to -0.57)

Viet Nam	41585 (36380 to 47477)	116.55 (102.43 to 133.75)	90172 (78824 to 102840)	110.09 (96.21 to 126.57)	-0.23 (-0.28 to -0.19)
Kazakhstan	11933 (10475 to 13665)	113.6 (99.49 to 130.25)	15444 (13387 to 17642)	111.1 (96.76 to 126.81)	-0.04 (-0.14 to 0.06)
Australia	24675 (21656 to 27883)	120.3 (106.3 to 134.67)	52191 (45954 to 58772)	102.66 (90.27 to 115.36)	-0.44 (-0.52 to -0.37)
Tonga	52 (45 to 60)	115.12 (100.05 to 132.42)	84 (73 to 97)	111.8 (97.12 to 128.75)	-0.04 (-1.46 to 1.4)
Fiji	266 (225 to 307)	112.81 (97.29 to 130.02)	582 (495 to 672)	110.65 (95.5 to 127.91)	-0.09 (-0.67 to 0.49)
Greece	19618 (16834 to 22921)	124.1 (107.53 to 142.61)	37087 (31838 to 42954)	119.17 (103.16 to 136.72)	-0.15 (-0.25 to -0.04)
Taiwan (Province of China)	11457 (9903 to 13141)	90.76 (78.7 to 104.03)	43204 (36980 to 48565)	98.1 (83.69 to 110.2)	0.48 (0.38 to 0.57)
Myanmar	20696 (17941 to 23619)	121.36 (106.48 to 137.66)	44028 (38392 to 49952)	113.09 (99.06 to 128.82)	-0.25 (-0.31 to -0.18)
Cyprus	878 (744 to 1037)	121.53 (105.52 to 138.93)	2459 (2096 to 2871)	117.37 (101.52 to 134.87)	-0.19 (-0.68 to 0.31)
Bulgaria	12362 (10399 to 14581)	115.07 (100.11 to 132.17)	17299 (14848 to 20198)	112.43 (97.8 to 128.73)	-0.09 (-0.24 to 0.06)
Hungary	15846 (13689 to 18464)	112.25 (98.26 to 128.61)	24331 (21065 to 27869)	111.47 (96.69 to 127.63)	-0.01 (-0.11 to 0.1)
Serbia	9626 (8222 to 11051)	111.52 (97.2 to 127.83)	19543 (16949 to 22543)	111.65 (96.91 to 128.06)	0.03 (-0.08 to 0.15)
Mongolia	948 (823 to 1083)	114.31 (100.16 to 130.48)	1819 (1599 to 2051)	115.83 (101.21 to 132.52)	0.1 (-0.22 to 0.43)
Trinidad and Tobago	786 (682 to 904)	100.44 (88.01 to 114.8)	1821 (1586 to 2070)	98.6 (85.73 to 112.51)	-0.06 (-0.44 to 0.31)
Marshall Islands	12 (10 to 14)	107.18 (92.38	21 (17 to 24)	103.94 (89.43	-0.59 (-3.7 to 2.63)

		to 123.7)		to 120.5)	
Montenegro	682 (593 to 782)	114.96 (100.51 to 131.88)	1006 (863 to 1163)	111.66 (97.13 to 128.15)	-0.17 (-0.6 to 0.26)
Cambodia	3785 (3277 to 4314)	116.54 (101.62 to 132.77)	10162 (8838 to 11662)	112.21 (97.73 to 128.37)	-0.17 (-0.32 to -0.02)
Sri Lanka	9699 (8465 to 11058)	110.9 (96.53 to 126.63)	25786 (22299 to 29882)	107.46 (93.3 to 123.54)	-0.13 (-0.23 to -0.04)
Romania	27277 (23253 to 31597)	111.59 (96.91 to 127.8)	46175 (39788 to 53464)	111.32 (96.82 to 127.73)	-0.03 (-0.11 to 0.04)
Armenia	2585 (2259 to 2947)	114.47 (99.79 to 130.46)	4930 (4260 to 5655)	114 (99.56 to 130.25)	0.01 (-0.2 to 0.22)
Slovenia	2750 (2387 to 3154)	109.95 (95.52 to 125.95)	5733 (4981 to 6582)	110.51 (96.63 to 126.67)	0.07 (-0.15 to 0.3)
Papua New Guinea	1332 (1143 to 1512)	119.96 (105.09 to 137.12)	3500 (3026 to 3981)	112.89 (97.86 to 129.7)	-0.26 (-0.52 to -0.01)
Turkmenistan	1690 (1483 to 1919)	112.46 (98.71 to 127.9)	3296 (2869 to 3741)	107.05 (93.39 to 122.99)	-0.19 (-0.42 to 0.04)
Czechia	15203 (13105 to 17550)	112.36 (97.87 to 128.36)	26327 (22779 to 30305)	111.23 (96.31 to 127.59)	-0.04 (-0.14 to 0.05)
Belgium	23042 (20167 to 26295)	136.46 (120.43 to 154.4)	34245 (29719 to 39179)	121.59 (105.24 to 139.55)	-0.4 (-0.5 to -0.3)
United Arab Emirates	345 (297 to 398)	123.25 (107.14 to 142.51)	2324 (1963 to 2691)	113.49 (98.05 to 131.08)	-0.23 (-0.71 to 0.25)
Lithuania	5078 (4393 to 5849)	111.58 (97.39 to 128.02)	7771 (6694 to 9018)	112.73 (98.22 to 129.2)	0.05 (-0.14 to 0.24)
Austria	16276 (14023 to 18889)	123.65 (107.51 to 141.53)	25291 (21751 to 29369)	116.09 (99.55 to 133.87)	-0.26 (-0.38 to -0.14)

Russian Federation	190282 (164523 to 219606)	117.56 (102.69 to 134.45)	279806 (243531 to 319869)	115.89 (101.23 to 131.98)	-0.06 (-0.08 to -0.03)
Slovakia	6446 (5589 to 7369)	111.96 (97.71 to 127.76)	10646 (9281 to 12234)	110.87 (97.08 to 127)	-0.05 (-0.19 to 0.1)
Latvia	4060 (3525 to 4677)	114.12 (99.75 to 130.94)	5467 (4733 to 6277)	114.97 (99.97 to 131)	0.07 (-0.15 to 0.29)
Republic of Korea	29055 (25309 to 32819)	126.55 (111.98 to 143.2)	117489 (102910 to 133127)	124.63 (109.44 to 140.89)	0.08 (-0.02 to 0.18)
Singapore	1709 (1490 to 1914)	91.19 (80.55 to 101.93)	7764 (6988 to 8584)	94.88 (85.54 to 104.85)	0.1 (-0.11 to 0.3)
Japan	200071 (174105 to 229289)	115.42 (100.73 to 131.35)	576270 (503457 to 662794)	117.23 (102.05 to 133.68)	0.28 (0.18 to 0.37)
France	94247 (84334 to 103887)	98.87 (89.02 to 109)	163141 (143062 to 184345)	93.63 (82.25 to 105.56)	-0.16 (-0.21 to -0.12)
North Macedonia	1748 (1524 to 1991)	111.77 (97.43 to 128.12)	3033 (2587 to 3532)	111.12 (96.41 to 127.65)	-0.01 (-0.26 to 0.25)
Israel	6189 (5277 to 7208)	119.68 (103.31 to 137.43)	15408 (13247 to 17722)	113.13 (97.01 to 130.26)	-0.23 (-0.38 to -0.08)
India	257735 (224227 to 293550)	79.04 (68.33 to 90.3)	749490 (648646 to 856823)	78.92 (68.29 to 90.58)	-0.14 (-0.16 to -0.12)
Belarus	14456 (12531 to 16564)	115.82 (100.91 to 132.62)	19115 (16467 to 21917)	116.35 (101.03 to 133.43)	0.03 (-0.07 to 0.14)
Republic of Moldova	3958 (3414 to 4538)	110.9 (96.49 to 126.27)	6801 (5910 to 7736)	111.97 (97.12 to 128.21)	0.03 (-0.15 to 0.22)
Brunei Darussalam	81 (70 to 94)	101.78 (87.9 to 117.59)	237 (205 to 273)	101.56 (88.37 to 116.86)	0.07 (-1.31 to 1.47)
Estonia	2264 (1941 to 2617)	113.71 (98.59 to 130.74)	3662 (3185 to 4204)	112.78 (98.89 to 128.28)	0.02 (-0.26 to 0.3)

Ukraine	78566 (67889 to 90270)	119.02 (103.91 to 135.64)	93467 (80738 to 107940)	115.57 (100.64 to 132.68)	-0.11 (-0.16 to -0.07)
Andorra	71 (60 to 81)	121.02 (104.88 to 138.58)	190 (164 to 218)	114.28 (98.41 to 131.55)	-0.15 (-1.44 to 1.17)
Luxembourg	578 (491 to 675)	100.97 (86.52 to 116.93)	1033 (880 to 1191)	87.36 (74.31 to 101.13)	-0.44 (-1 to 0.11)
Spain	66079 (57996 to 73532)	112.03 (98.68 to 123.28)	123770 (107148 to 142152)	102.5 (88.99 to 116.45)	-0.24 (-0.31 to -0.17)
Pakistan	39180 (33966 to 44720)	84.21 (73.14 to 96.59)	67818 (58773 to 77287)	78.8 (67.99 to 90.38)	-0.25 (-0.3 to -0.2)
Finland	9390 (8168 to 10732)	120.98 (105.76 to 137.25)	16662 (14213 to 19329)	108.45 (92.52 to 124.81)	-0.33 (-0.48 to -0.18)
New Zealand	5268 (4565 to 6055)	127.8 (111.1 to 145.96)	11097 (9639 to 12753)	120.84 (104.92 to 138.57)	-0.23 (-0.39 to -0.07)
Germany	204241 (182945 to 226402)	145.4 (130.74 to 160.44)	339825 (299328 to 382749)	142.13 (124.57 to 159.72)	0.12 (-0.12 to 0.35)
Italy	111576 (94488 to 128892)	115.83 (98.69 to 132.49)	248809 (215069 to 285482)	134.76 (116.74 to 153.65)	-0.05 (-0.29 to 0.2)
Malta	515 (443 to 596)	122.04 (105.66 to 140)	1295 (1113 to 1493)	113.91 (98.03 to 130.83)	-0.22 (-0.78 to 0.35)
Netherlands	27847 (24500 to 30791)	127.03 (112.09 to 139.74)	48272 (42168 to 54730)	121.79 (106.84 to 137.86)	0.09 (-0.08 to 0.26)
Uruguay	4635 (4017 to 5293)	113.1 (98.89 to 128.49)	7017 (6124 to 8053)	107.09 (93.07 to 121.78)	-0.23 (-0.43 to -0.04)
Portugal	16587 (14199 to 19312)	118.79 (102.75 to 136.06)	35666 (30670 to 41514)	116.97 (100.45 to 134.76)	-0.02 (-0.13 to 0.08)
Canada	53906 (48456 to 59760)	155.64 (140.55 to 171.87)	105264 (93819 to 117160)	132.4 (118.02 to 147.29)	-0.59 (-0.67 to -0.51)

Belize	95 (83 to 109)	100.29 (87.44 to 114.78)	244 (214 to 276)	97.17 (84.64 to 110.79)	-0.11 (-1.07 to 0.85)
Dominica	59 (51 to 68)	99.29 (86.4 to 113.22)	74 (64 to 84)	96.57 (83.47 to 110.4)	-0.2 (-1.99 to 1.63)
Norway	10767 (9395 to 12373)	134.49 (117.52 to 152.85)	13149 (11352 to 15113)	114.3 (98.69 to 131.41)	-0.6 (-0.75 to -0.45)
Ireland	5187 (4445 to 6032)	122.19 (105.76 to 140.41)	9579 (8205 to 11080)	111.99 (95.75 to 128.99)	-0.3 (-0.48 to -0.12)
Switzerland	15095 (13093 to 17295)	124.5 (108.87 to 142.44)	24776 (21498 to 28441)	113.27 (97.88 to 129.61)	-0.38 (-0.5 to -0.26)
United Kingdom	115427 (99727 to 133198)	113.91 (99.28 to 130.08)	164947 (142721 to 190062)	107.93 (93.37 to 123.89)	-0.18 (-0.27 to -0.08)
Sweden	24442 (21503 to 27653)	134.31 (118.97 to 151.02)	33511 (29061 to 38427)	126.32 (109.55 to 144.06)	-0.02 (-0.19 to 0.16)
Barbados	330 (285 to 385)	98.36 (86.39 to 112.47)	497 (433 to 573)	94.55 (82.58 to 109.16)	-0.14 (-0.83 to 0.56)
Dominican Republic	3358 (2922 to 3844)	100 (87.38 to 113.63)	9479 (8275 to 10748)	99.53 (86.68 to 113.07)	-0.16 (-0.32 to 0)
Antigua and Barbuda	58 (50 to 67)	97.18 (84.42 to 111.33)	88 (76 to 101)	95.08 (82.7 to 109.64)	0.09 (-1.38 to 1.58)
Cuba	9728 (8493 to 11029)	93.06 (81.15 to 105.22)	19278 (16865 to 21941)	91.41 (79.7 to 103.65)	-0.22 (-0.33 to -0.1)
Chile	10283 (9008 to 11672)	109.81 (96.22 to 123.58)	28466 (24777 to 32535)	107.87 (93.91 to 123.11)	-0.03 (-0.13 to 0.07)
United States of America	492880 (429701 to 564818)	138.35 (120.88 to 157.03)	822911 (718803 to 935112)	131.29 (113.93 to 149.6)	-0.14 (-0.22 to -0.07)
Argentina	34827 (30211 to 39910)	112.03 (97.43 to 128.19)	62825 (54326 to 72188)	106.73 (92.2 to 122.29)	-0.2 (-0.26 to -0.14)
Grenada	83 (72 to 96)	99.56 (86.98 to 114.23)	92 (79 to 105)	98.02 (85.92 to 112.22)	0.02 (-1.3 to 1.36)

Guyana	292 (254 to 332)	97.25 (83.98 to 111.33)	481 (419 to 548)	96.25 (83.56 to 110.32)	-0.05 (-0.63 to 0.53)
Haiti	2321 (2000 to 2642)	101.59 (88.48 to 115.53)	4672 (4066 to 5354)	94.82 (82.42 to 108.66)	-0.24 (-0.44 to -0.04)
Jamaica	2182 (1901 to 2498)	110.25 (96.1 to 125.59)	3490 (3046 to 3974)	104.77 (91.01 to 120.07)	-0.22 (-0.47 to 0.03)
Saint Vincent and the Grenadines	69 (60 to 80)	101 (87.52 to 115.23)	128 (111 to 146)	96.56 (84.19 to 110.27)	-0.19 (-1.48 to 1.11)
Democratic Republic of the Congo	13164 (11342 to 15031)	126.78 (110.3 to 143.99)	29847 (26225 to 33722)	126.99 (111.77 to 144.57)	-0.05 (-0.14 to 0.03)
Bahamas	136 (119 to 156)	97.08 (84.51 to 110.72)	326 (285 to 372)	95.28 (82.97 to 109.64)	0.01 (-0.78 to 0.81)
Suriname	240 (209 to 272)	104.75 (91.6 to 119.86)	580 (506 to 663)	100.19 (87.41 to 115.47)	-0.18 (-0.79 to 0.44)
Saint Lucia	81 (69 to 94)	99.01 (86.18 to 113.38)	223 (192 to 255)	95.98 (82.99 to 109.74)	-0.25 (-1.36 to 0.88)
Costa Rica	1972 (1721 to 2256)	113.01 (98.53 to 129.41)	6143 (5378 to 6985)	110.62 (95.96 to 126.87)	-0.06 (-0.27 to 0.15)
Bolivia (Plurinational State of)	2131 (1841 to 2439)	82.56 (71.74 to 94.39)	6052 (5222 to 6916)	82.05 (71.22 to 94.16)	0.02 (-0.18 to 0.21)
Peru	8933 (7783 to 10125)	79.62 (69.34 to 90.7)	25917 (22445 to 29526)	78.28 (67.57 to 89.77)	-0.1 (-0.2 to 0)
El Salvador	3296 (2903 to 3732)	110.78 (97.12 to 126.07)	7619 (6700 to 8708)	112.22 (98.14 to 128.45)	0.07 (-0.11 to 0.25)
Nicaragua	1601 (1403 to 1813)	114.78 (100.41 to 130.39)	4883 (4277 to 5562)	114.62 (100.58 to 131.05)	0.02 (-0.2 to 0.24)
Brazil	98068 (85802 to 111118)	129.04 (113.25 to 146.32)	305421 (269292 to 345696)	127.08 (112.01 to 144.66)	-0.06 (-0.12 to 0)
Madagascar	3808 (3315 to 4320)	104.43 (91.35 to 119.44)	6743 (5808 to 7654)	99.76 (86.8 to 114.13)	-0.14 (-0.29 to 0.01)

Ecuador	4011 (3471 to 4605)	82.43 (71.41 to 94.77)	12517 (10756 to 14473)	81.16 (69.99 to 93.4)	-0.02 (-0.16 to 0.12)
Venezuela (Bolivarian Republic of)	11339 (9902 to 12877)	128.24 (111.91 to 145.3)	34325 (30254 to 38771)	124.37 (108.4 to 141.62)	-0.11 (-0.19 to -0.03)
Colombia	17564 (15423 to 19922)	113.29 (98.82 to 129.37)	63668 (55802 to 72058)	112.13 (97.75 to 127.51)	-0.02 (-0.09 to 0.05)
Guatemala	2921 (2552 to 3331)	113.43 (99.15 to 129.47)	10938 (9620 to 12428)	112.54 (98.73 to 127.7)	-0.02 (-0.19 to 0.16)
Honduras	2026 (1771 to 2313)	114.77 (100.53 to 131.28)	5675 (4919 to 6495)	113.19 (98.75 to 129.15)	-0.04 (-0.24 to 0.16)
Mexico	37949 (32951 to 43436)	103.1 (89.53 to 117.63)	110467 (96168 to 125494)	97.19 (84.27 to 111.26)	-0.14 (-0.19 to -0.1)
Panama	1607 (1404 to 1825)	110.15 (96 to 125.54)	4943 (4336 to 5638)	108.64 (94.58 to 124.54)	-0.05 (-0.28 to 0.19)
Iraq	9666 (8465 to 10949)	135.31 (118.13 to 154.01)	22204 (19382 to 25184)	129.62 (113.05 to 148.98)	-0.18 (-0.27 to -0.09)
Lebanon	2570 (2255 to 2921)	140.43 (123.12 to 159.83)	9167 (8003 to 10453)	140.48 (122.53 to 159.62)	0 (-0.18 to 0.18)
Eritrea	611 (526 to 692)	109.92 (95.74 to 125.59)	1735 (1503 to 1975)	104.21 (90.44 to 119.18)	-0.14 (-0.55 to 0.27)
Paraguay	2629 (2303 to 2975)	122.93 (107.39 to 139.55)	6135 (5388 to 6970)	115.77 (101.31 to 132.33)	-0.21 (-0.39 to -0.03)
Bahrain	144 (124 to 164)	133.15 (116.31 to 151.71)	692 (594 to 788)	130.61 (114.78 to 148.7)	0.18 (-1.05 to 1.43)
Jordan	1298 (1131 to 1476)	132.21 (114.69 to 151.07)	7243 (6314 to 8274)	134.51 (116.99 to 153.81)	0.1 (-0.11 to 0.32)
Algeria	13226 (11355 to 15365)	133.66 (116.97 to 152.44)	37174 (32167 to 43066)	128.59 (112.65 to 146.91)	-0.13 (-0.25 to -0.02)

Egypt	23384 (20531 to 26090)	128.38 (114.25 to 144.51)	50931 (44459 to 57309)	126.46 (111.45 to 144.07)	-0.07 (-0.13 to 0)
Iran (Islamic Republic of)	25143 (21708 to 28637)	137.09 (120.6 to 155.91)	87937 (77508 to 99879)	133.73 (117.53 to 151.85)	-0.05 (-0.11 to 0)
Libya	2264 (1997 to 2549)	136.08 (119.46 to 154.53)	5287 (4619 to 5998)	130.47 (114.04 to 148.13)	-0.15 (-0.34 to 0.03)
Kuwait	603 (530 to 678)	136.6 (119.53 to 155.03)	2814 (2484 to 3155)	131.46 (115.44 to 149.34)	-0.11 (-0.42 to 0.19)
Morocco	17075 (14858 to 19361)	136.09 (118.85 to 155.06)	36514 (31637 to 41831)	127.85 (111.31 to 146.15)	-0.24 (-0.31 to -0.17)
Palestine	983 (859 to 1118)	136.85 (120.04 to 155.15)	2402 (2103 to 2729)	131.55 (115.83 to 150.08)	-0.18 (-0.47 to 0.12)
Saudi Arabia	5686 (4959 to 6481)	127.56 (111.24 to 146.12)	13064 (11315 to 14779)	120.44 (104.42 to 138.45)	-0.21 (-0.32 to -0.1)
Malawi	2832 (2442 to 3230)	105.4 (91.67 to 120.77)	5280 (4599 to 6027)	104.71 (90.82 to 119.89)	-0.03 (-0.21 to 0.16)
Qatar	81 (70 to 91)	128.19 (111.46 to 146.56)	640 (553 to 721)	126.56 (110.1 to 145.4)	0.14 (-1.09 to 1.38)
Oman	666 (582 to 753)	131.81 (115.59 to 150.61)	1619 (1401 to 1863)	124.36 (108.14 to 143.21)	-0.13 (-0.47 to 0.2)
Turkey	42666 (37375 to 48311)	147.16 (128.67 to 166.63)	117991 (102838 to 133396)	139.1 (120.57 to 158.16)	-0.22 (-0.27 to -0.18)
Syrian Arab Republic	5648 (4959 to 6393)	138.62 (121.31 to 157.84)	13051 (11335 to 14936)	129.77 (113.5 to 147.95)	-0.22 (-0.35 to -0.1)
Tunisia	6004 (5202 to 6825)	141.66 (124.04 to 160.74)	15845 (13951 to 18011)	134.32 (117.99 to 153.04)	-0.19 (-0.32 to -0.07)
Yemen	5035 (4373 to 5725)	143.07 (125.88 to 162.62)	13410 (11724 to 15191)	133.11 (117.14 to 151.66)	-0.24 (-0.37 to -0.11)

Afghanistan	6914 (5989 to 7884)	132.98 (116.42 to 151.73)	8764 (7688 to 10026)	130.94 (115.12 to 149.09)	-0.06 (-0.2 to 0.07)
Seychelles	64 (56 to 74)	113.24 (98.52 to 129.83)	108 (94 to 124)	109.25 (94.47 to 126.6)	-0.36 (-1.61 to 0.91)
Somalia	1509 (1313 to 1697)	107.43 (93.77 to 122.3)	3590 (3086 to 4105)	106.72 (93.34 to 121.51)	-0.03 (-0.27 to 0.21)
Bangladesh	30924 (26840 to 35296)	82.3 (70.98 to 94.25)	92254 (79987 to 105807)	79.47 (68.72 to 90.87)	-0.11 (-0.17 to -0.06)
Bhutan	138 (120 to 157)	83.15 (72.17 to 95.37)	415 (360 to 475)	77.45 (67.36 to 88.68)	-0.24 (-0.98 to 0.5)
Nepal	6014 (5254 to 6820)	90.94 (79.26 to 103.61)	14856 (12919 to 16960)	81.31 (70.51 to 93.7)	-0.44 (-0.56 to -0.33)
Angola	3105 (2700 to 3510)	127.18 (111.54 to 144.47)	9171 (7979 to 10423)	124.21 (109.07 to 141.23)	-0.06 (-0.23 to 0.11)
Central African Republic	923 (791 to 1047)	134.26 (118.33 to 152.43)	1622 (1391 to 1837)	130.87 (114.21 to 148.33)	-0.14 (-0.49 to 0.2)
Congo	908 (783 to 1044)	124.09 (108.43 to 141.67)	2079 (1886 to 2269)	120.8 (109.94 to 132.53)	-0.05 (-0.47 to 0.38)
Equatorial Guinea	167 (146 to 190)	129.07 (112.91 to 146.07)	431 (377 to 490)	124.45 (108.79 to 141.57)	-0.11 (-0.84 to 0.63)
Gabon	586 (509 to 669)	125.64 (109.37 to 143.64)	911 (794 to 1042)	123.01 (107.72 to 140.92)	-0.12 (-0.54 to 0.29)
Burundi	1863 (1608 to 2126)	108.15 (94.65 to 122.98)	3237 (2823 to 3672)	100.5 (88.07 to 114.87)	-0.2 (-0.43 to 0.04)
Djibouti	94 (82 to 107)	108.76 (95.11 to 123.96)	402 (347 to 454)	104.02 (90.57 to 118.65)	-0.17 (-0.97 to 0.64)
Coted'Ivoire	2044 (1770 to 2322)	79.52 (68.92 to 91.37)	5598 (4850 to 6401)	76.63 (66.06 to 88.28)	-0.12 (-0.31 to 0.06)
Comoros	147 (127 to 168)	104.63 (91.44 to 118.88)	381 (330 to 432)	100.96 (88.05 to 115.39)	-0.1 (-0.85 to 0.66)

Ethiopia	13750 (11888 to 15616)	109.29 (95.57 to 124.26)	34226 (30050 to 38961)	102.87 (90.07 to 116.73)	-0.14 (-0.24 to -0.05)
Kenya	7010 (6119 to 7949)	107.07 (93.41 to 121.96)	16494 (14435 to 18732)	105.14 (91.55 to 119.62)	-0.11 (-0.21 to 0)
Mauritius	660 (569 to 758)	112.09 (97.53 to 128.76)	1883 (1629 to 2139)	111.73 (96.55 to 127.55)	0.04 (-0.3 to 0.39)
Mozambique	4509 (3902 to 5110)	107.36 (94.05 to 122.84)	7516 (6505 to 8549)	104.33 (90.49 to 119.04)	-0.12 (-0.26 to 0.02)
Rwanda	1968 (1701 to 2235)	106.36 (93 to 121.52)	4609 (4024 to 5193)	106.57 (93.24 to 120.76)	0.06 (-0.16 to 0.28)
United Republic of Tanzania	8601 (7577 to 9681)	105.95 (93.42 to 119.2)	18873 (16815 to 21095)	98.48 (87.78 to 110.14)	-0.24 (-0.34 to -0.14)
Botswana	424 (365 to 483)	107.65 (93.62 to 122.91)	1062 (924 to 1218)	103.94 (90.71 to 119.61)	-0.07 (-0.53 to 0.4)
Uganda	5022 (4361 to 5706)	104.06 (90.86 to 118.38)	10731 (9352 to 12174)	102.68 (89.05 to 117.37)	-0.05 (-0.18 to 0.08)
Zambia	2064 (1795 to 2346)	101.93 (88.76 to 115.9)	4723 (4112 to 5326)	102.65 (89.99 to 117.22)	0.04 (-0.16 to 0.25)
Lesotho	769 (669 to 876)	109.88 (95.45 to 125.25)	791 (682 to 906)	108.92 (94.83 to 124.74)	-0.04 (-0.43 to 0.35)
South Africa	20560 (17955 to 23217)	113.1 (98.7 to 129.16)	39715 (34529 to 45264)	107.78 (93.63 to 122.59)	-0.13 (-0.19 to -0.06)
Eswatini	212 (184 to 241)	104.32 (90.99 to 119.37)	359 (310 to 411)	101.07 (88.65 to 115.8)	-0.11 (-0.77 to 0.55)
Zimbabwe	3214 (2773 to 3675)	105.39 (91.76 to 120.16)	4505 (3921 to 5133)	102.44 (89.41 to 116.93)	-0.06 (-0.24 to 0.11)
Namibia	489 (422 to 556)	106.73 (93.53 to 121.78)	1012 (884 to 1148)	103.46 (89.84 to 117.7)	-0.08 (-0.51 to 0.35)
Benin	1376 (1208 to 1566)	81.84 (71.81 to 93.16)	2909 (2552 to 3316)	75.85 (65.91 to 87.05)	-0.21 (-0.45 to 0.03)

Burkina Faso	2714 (2348 to 3111)	84.91 (73.58 to 97.02)	5394 (4695 to 6140)	79.8 (69.33 to 91.77)	-0.24 (-0.42 to -0.06)
Cameroon	2565 (2220 to 2919)	79.31 (68.75 to 90.99)	6433 (5588 to 7316)	75.45 (65.39 to 86.37)	-0.19 (-0.36 to -0.02)
Ghana	3390 (2934 to 3845)	76.62 (66 to 88)	8608 (7446 to 9814)	75.09 (64.72 to 86.42)	-0.06 (-0.21 to 0.09)
Cabo Verde	209 (180 to 242)	81.92 (70.75 to 93.85)	319 (276 to 363)	78.13 (67.85 to 89.4)	-0.26 (-1.03 to 0.52)
Chad	1931 (1675 to 2212)	83.52 (72.31 to 95.78)	3052 (2649 to 3472)	76.45 (66.54 to 87.28)	-0.31 (-0.53 to -0.09)
Gambia	216 (188 to 247)	83.67 (72.87 to 95.98)	573 (493 to 658)	77.92 (67.57 to 89.88)	-0.27 (-0.86 to 0.33)
Niger	1526 (1319 to 1732)	82.8 (71.98 to 95.34)	4296 (3712 to 4924)	78.36 (68.05 to 90.06)	-0.17 (-0.39 to 0.05)
Guinea-Bissau	203 (174 to 231)	78.72 (67.95 to 90.63)	330 (283 to 376)	77.05 (66.66 to 89.04)	-0.02 (-0.73 to 0.69)
Guinea	2309 (2006 to 2617)	83.04 (72.5 to 94.9)	3372 (2920 to 3850)	78.39 (67.86 to 90.01)	-0.24 (-0.44 to -0.03)
Liberia	694 (596 to 796)	77.6 (67.38 to 88.97)	1101 (956 to 1253)	75.16 (64.95 to 86.14)	-0.14 (-0.51 to 0.23)
Mali	2169 (1879 to 2470)	81.5 (71.35 to 93.08)	4712 (4072 to 5355)	78.55 (68.4 to 89.84)	-0.12 (-0.32 to 0.07)
Senegal	2055 (1771 to 2337)	82.01 (71.31 to 94)	4513 (3919 to 5167)	76.64 (66.53 to 88.26)	-0.25 (-0.45 to -0.06)
Nigeria	25694 (22282 to 29177)	75.1 (65.04 to 85.62)	45312 (39635 to 51186)	68.83 (59.58 to 78.6)	-0.23 (-0.28 to -0.17)
Sao Tome and Principe	42 (36 to 48)	78.02 (67.66 to 89.88)	60 (52 to 70)	73.4 (63.08 to 84.79)	-0.32 (-1.92 to 1.3)
Mauritania	652 (559 to 745)	82.98 (71.62 to 95.11)	1316 (1142 to 1500)	77.53 (67.33 to 88.83)	-0.2 (-0.57 to 0.17)
Sierra Leone	1391 (1208 to 1594)	83.45 (72.82 to 94.85)	2201 (1908 to 2531)	79.15 (68.54 to 91.02)	-0.2 (-0.47 to 0.06)

Togo	709 (619 to 805)	80.67 (69.92 to 92.56)	1900 (1634 to 2158)	78.26 (67.73 to 90)	-0.13 (-0.44 to 0.19)
American Samoa	18 (15 to 21)	111.48 (96.23 to 128.37)	41 (35 to 47)	109.14 (93.39 to 125.47)	-0.44 (-2.8 to 1.98)
Greenland	32 (27 to 36)	139.66 (121.08 to 159.9)	69 (60 to 79)	135.63 (117.63 to 156.11)	-0.33 (-2.32 to 1.7)
Guam	60 (51 to 70)	109.68 (94.45 to 127.52)	242 (210 to 275)	108.97 (93.85 to 125.54)	0.09 (-1.3 to 1.51)
Bermuda	59 (50 to 68)	99.71 (86.05 to 114.57)	150 (130 to 173)	98.24 (84.8 to 112.71)	-0.51 (-2.13 to 1.13)
Monaco	108 (93 to 126)	122.71 (106.16 to 141.01)	136 (116 to 157)	113.72 (97.81 to 131.33)	-0.35 (-2.12 to 1.44)
Cook Islands	11 (10 to 13)	111.77 (95.49 to 129.15)	28 (24 to 32)	109.53 (93.6 to 126.15)	-0.39 (-3.55 to 2.86)
Nauru	3 (3 to 4)	111.9 (96.21 to 129.18)	4 (4 to 5)	113.97 (98.71 to 131.23)	NA
Northern Mariana Islands	11 (9 to 13)	109.43 (93.88 to 127.66)	38 (32 to 44)	107.94 (92.46 to 125.17)	-0.88 (-3.68 to 2.01)
Palau	8 (7 to 10)	106.89 (91.51 to 124.12)	16 (13 to 18)	103.98 (88.42 to 120.53)	-0.66 (-4.29 to 3.11)
Puerto Rico	3779 (3263 to 4354)	99.76 (87.12 to 114.02)	8775 (7670 to 10036)	97.8 (84.88 to 112.14)	-0.09 (-0.28 to 0.1)
Niue	3 (2 to 3)	110.15 (95.02 to 127.07)	2 (2 to 3)	107.28 (91.33 to 123.83)	0.08 (-8.04 to 8.92)
San Marino	52 (45 to 59)	123.67 (108.13 to 141.19)	106 (92 to 122)	110.9 (96.02 to 127.71)	-0.36 (-2.42 to 1.75)
United States Virgin Islands	69 (59 to 79)	97.13 (84.11 to 111.29)	181 (156 to 212)	94.57 (82.02 to 109.17)	-0.12 (-1.79 to 1.59)
Tuvalu	5 (5 to 6)	115.76 (100.06 to 132.92)	9 (8 to 11)	113.41 (97.97 to 130.03)	NA

Saint Kitts and Nevis	35 (30 to 41)	94.4 (82.44 to 108.43)	48 (42 to 56)	93.38 (80.85 to 107.47)	0.03 (-2.29 to 2.4)
South Sudan	2028 (1756 to 2318)	104.61 (91.25 to 119.47)	2565 (2229 to 2899)	99.16 (85.85 to 113.38)	-0.13 (-0.35 to 0.1)
Sudan	10295 (8913 to 11733)	133.97 (117.29 to 152.94)	18538 (16236 to 21018)	125.81 (110.08 to 143.27)	-0.23 (-0.33 to -0.13)

Abbreviations: ASIR: age-standardized incidence rate; APC: age-period-cohort; UI: uncertainty intervals; CI: confidence interval.

Table S2. Numbers of cases and incidence rates of AD and other dementias at global and national levels in 2022 and 2036.

location	2022		2036	
	Number (95%UI)	ASIR (95%UI)	Number (95%UI)	ASIR (95%UI)
Global	10352741 (10136930 to 10568551)	360.09 (352.59 to 367.6)	19117304 (14380594 to 23854014)	418.92 (315.03 to 522.8)
Andorra	186 (71 to 308)	338.52 (130.18 to 562.65)	297 (33 to 575)	327.73 (34.94 to 652.54)
Australia	52812 (49919 to 55705)	307.65 (290.1 to 325.2)	81658 (53169 to 110146)	312.89 (202.89 to 422.88)
Austria	26304 (24546 to 28062)	345.9 (321.08 to 370.71)	35298 (23598 to 46998)	340.81 (226.05 to 455.58)
Belgium	34876 (32787 to 36964)	362.65 (339.43 to 385.88)	46079 (31194 to 60964)	355.29 (238.91 to 471.66)
Bermuda	153 (51 to 263)	294.59 (96.3 to 517.42)	253 (22 to 504)	305.95 (22.64 to 640.17)
Brunei Darussalam	281 (123 to 443)	305.09 (137.96 to 477.4)	637 (109 to 1178)	323.26 (55.47 to 598.33)
Canada	109566 (103854 to 115278)	391.82 (370.8 to 412.83)	148212 (92497 to 203927)	346.12 (214.9 to 477.34)
Cyprus	2774 (2258 to 3290)	351.42 (283.74 to 419.1)	4514 (2253 to 6778)	351.56 (172.72 to 530.87)
Czechia	27105 (25294 to 28917)	332.33 (308.65 to 356.01)	38483 (25822 to 51143)	338.27 (225.21 to 451.33)
Denmark	11673 (10554 to 12792)	252.04 (225.93 to 278.15)	16299 (9548 to 23049)	261.9 (151.27 to 372.53)
Estonia	3746 (3172 to 4321)	335.6 (278.43 to 392.76)	4728 (2527 to 6932)	335.29 (173.7 to 497.47)
Finland	16557 (15213 to 17902)	323.64 (294.75 to 352.54)	22119 (14225 to 30013)	320.93 (203.5 to 438.37)
France	168549 (162122 to 174977)	280.19 (268.89 to 291.49)	235776 (156536 to 315017)	285.33 (188.38 to 382.29)
Germany	353484 (325081 to 381887)	436.52 (401.25 to 471.79)	447886 (286461 to 609311)	454.74 (290.15 to 619.34)
Guam	197 (73 to 327)	323.77 (122.4 to 535.44)	343 (47 to 653)	337.73 (43.79 to 653.33)
Iceland	812 (555 to 1073)	367.26 (247.21 to 490.21)	1345 (508 to 2192)	380.34 (135.67 to 630.73)

Ireland	9849 (8837 to 10862)	332.82 (297.49 to 368.15)	15400 (9467 to 21332)	320.66 (195.18 to 446.13)
Japan	591182 (562012 to 620352)	357.13 (339.09 to 375.17)	607101 (378287 to 835915)	301.28 (187.81 to 414.75)
Kuwait	3408 (2778 to 4039)	391.71 (324.15 to 459.26)	9556 (4864 to 14247)	401.53 (205.51 to 597.55)
Latvia	5554 (4847 to 6260)	342.93 (294.64 to 391.22)	6440 (3734 to 9149)	347.51 (196.78 to 498.6)
Lithuania	7874 (7007 to 8740)	338.17 (297.16 to 379.17)	9487 (5430 to 13543)	357.1 (200.93 to 513.27)
Luxembourg	1024 (729 to 1320)	260.48 (182.89 to 338.92)	1595 (626 to 2571)	265.39 (100.48 to 432.68)
Netherlands	50504 (46999 to 54009)	368.12 (341.61 to 394.63)	73281 (47869 to 98693)	370.99 (240.96 to 501.03)
New Zealand	11406 (10300 to 12513)	360.99 (324.09 to 397.88)	17403 (10758 to 24048)	359.46 (219.39 to 499.53)
Norway	13259 (12071 to 14446)	341.69 (309.39 to 373.98)	20000 (12382 to 27618)	351.24 (215.16 to 487.31)
Puerto Rico	9014 (8050 to 9978)	291.75 (257.89 to 325.61)	13041 (7984 to 18099)	294.38 (176.48 to 412.28)
Qatar	978 (656 to 1299)	371.57 (232.98 to 510.59)	4167 (1418 to 6918)	383.75 (127.41 to 640.19)
Republic of Korea	129580 (122983 to 136178)	375.81 (356.43 to 395.2)	231530 (158094 to 304966)	370 (251.89 to 488.12)
Russian Federation	290911 (282369 to 299452)	346.24 (335.87 to 356.61)	411047 (307121 to 514974)	350.41 (261.36 to 439.46)
Saudi Arabia	16467 (14981 to 17953)	360.53 (328.18 to 392.89)	45950 (29056 to 62845)	353.51 (224.02 to 483.01)
Singapore	8092 (7131 to 9052)	287 (253.38 to 320.62)	18765 (10806 to 26724)	304.32 (173.98 to 434.65)
Slovakia	10922 (9851 to 11992)	331.4 (297.57 to 365.24)	16665 (10424 to 22905)	341.22 (211.47 to 470.96)
Slovenia	5805 (5056 to 6554)	329.78 (283.94 to 375.62)	8084 (4656 to 11514)	330.01 (185.54 to 474.66)

Sweden	34132 (31707 to 36557)	381.67 (352.61 to 410.74)	46817 (30870 to 62764)	400.25 (262.08 to 538.42)
Switzerland	25182 (23463 to 26900)	338.29 (313.62 to 362.96)	35537 (23501 to 47574)	329.53 (216.23 to 442.83)
Taiwan (Province of China)	43620 (40875 to 46366)	293.46 (274.86 to 312.06)	76049 (31494 to 120605)	307.41 (127.06 to 487.76)
United Arab Emirates	2922 (2349 to 3495)	350.94 (255.55 to 446.33)	11168 (5360 to 16975)	343.2 (155.75 to 530.64)
United Kingdom	170170 (162012 to 178328)	323.57 (307.61 to 339.53)	220882 (142722 to 299043)	311.8 (200.66 to 422.93)
United States of America	849780 (809675 to 889886)	391.85 (373.25 to 410.45)	1269354 (889446 to 1649262)	394.61 (276.1 to 513.11)
American Samoa	39 (0 to 95)	320.57 (0 to 814.12)	75 (0 to 192)	333.04 (0 to 883.7)
Antigua and Barbuda	101 (18 to 195)	275.64 (52.31 to 522.63)	176 (1 to 386)	287.3 (1.61 to 636.38)
Argentina	64704 (61603 to 67804)	318.81 (303.28 to 334.34)	88316 (62514 to 114118)	312.57 (220.9 to 404.24)
Bahamas	358 (182 to 538)	284.14 (147.45 to 422.8)	660 (154 to 1177)	293.55 (67.24 to 525.59)
Bahrain	1118 (764 to 1473)	387.42 (263.26 to 511.58)	3720 (1319 to 6125)	394.64 (137.93 to 651.86)
Barbados	518 (313 to 727)	281.85 (166.58 to 400.44)	757 (233 to 1290)	282.72 (80.49 to 492.62)
Belarus	19706 (18228 to 21183)	348.1 (321.21 to 374.98)	26929 (17899 to 35959)	360.15 (237.96 to 482.33)
Bosnia and Herzegovina	6951 (6126 to 7777)	333.24 (290.67 to 375.8)	9493 (5671 to 13316)	333.52 (196.54 to 470.5)
Bulgaria	18699 (17256 to 20141)	334.73 (306.64 to 362.82)	21461 (14071 to 28852)	332.74 (216.03 to 449.45)
Chile	29068 (27116 to 31019)	321.99 (300.1 to 343.88)	47194 (30986 to 63401)	315.91 (206.62 to 425.19)
Croatia	11342 (10265 to 12420)	336.68 (301.59 to 371.76)	14269 (8939 to 19599)	340.17 (210.11 to 470.23)
Dominica	98 (18 to 188)	285.51 (52.38 to 553.22)	134 (0 to 303)	315.11 (0.35 to 722.92)
Greece	36934 (34777 to 39092)	355.18 (332.41 to 377.95)	40768 (27777 to 53760)	342.21 (231.5 to 452.92)

Greenland	87 (12 to 173)	408.81 (58.93 to 818.15)	178 (0 to 397)	462.78 (0 to 1060.79)
Hungary	25043 (23328 to 26758)	332.8 (308.65 to 356.96)	32184 (21313 to 43055)	333.25 (218.9 to 447.59)
Israel	15805 (14468 to 17142)	337.92 (308.37 to 367.48)	25388 (16284 to 34492)	337.6 (215.13 to 460.06)
Italy	242418 (214678 to 270158)	384.05 (339.9 to 428.19)	335702 (186917 to 484487)	425.03 (235.94 to 614.11)
Jordan	7733 (6777 to 8689)	404.51 (353.69 to 455.34)	18165 (10315 to 26016)	418.77 (238.26 to 599.27)
Kazakhstan	16960 (15557 to 18364)	331.12 (303.57 to 358.67)	28324 (18393 to 38255)	332.85 (216.12 to 449.58)
Lebanon	8668 (7692 to 9643)	422.18 (374.73 to 469.63)	14830 (8921 to 20740)	418.7 (252.74 to 584.65)
Libya	5317 (4558 to 6077)	389.36 (335.05 to 443.67)	10196 (5819 to 14573)	392.66 (224.85 to 560.48)
Malaysia	27116 (25198 to 29035)	331.92 (308.33 to 355.5)	45001 (29387 to 60616)	302.65 (197.79 to 407.52)
Malta	1238 (924 to 1556)	340.17 (246.53 to 435.64)	1769 (759 to 2785)	341.3 (138.39 to 548.18)
Montenegro	1158 (841 to 1476)	331.46 (236.04 to 427.48)	1516 (628 to 2410)	333.39 (133.52 to 535.4)
North Macedonia	4229 (3567 to 4892)	334.57 (280.65 to 388.49)	6704 (3604 to 9804)	341.49 (181.05 to 501.97)
Northern Mariana Islands	50 (0 to 115)	327.65 (2.7 to 776.27)	122 (0 to 295)	352.55 (0 to 898.27)
Oman	2351 (1837 to 2864)	366.48 (289.75 to 443.22)	6630 (3051 to 10210)	365.74 (171.54 to 559.93)
Poland	92944 (89026 to 96862)	340.82 (325.89 to 355.76)	137373 (98418 to 176328)	341.73 (243.92 to 439.54)
Portugal	35910 (33784 to 38036)	348.61 (326.22 to 371)	45680 (31150 to 60210)	339.55 (229.65 to 449.46)
Romania	46786 (44271 to 49301)	332.89 (313.9 to 351.87)	60270 (41969 to 78572)	342.16 (236.99 to 447.33)

Serbia	17505 (16120 to 18890)	331.68 (302.59 to 360.76)	21304 (13953 to 28654)	333.63 (216.69 to 450.58)
Seychelles	112 (23 to 208)	327.4 (73.58 to 604.51)	184 (6 to 389)	333.6 (11.09 to 712.53)
Spain	124899 (117752 to 132045)	298.99 (281.37 to 316.62)	194894 (79106 to 310683)	351.14 (143.08 to 559.2)
Trinidad and Tobago	1950 (1519 to 2380)	293.87 (228.92 to 358.83)	3355 (1608 to 5105)	296.75 (139.5 to 454.53)
Turkey	145253 (139758 to 150748)	416.08 (400.47 to 431.68)	262089 (190143 to 334034)	410.01 (296.96 to 523.06)
Ukraine	94860 (90983 to 98737)	345.88 (331.19 to 360.56)	116198 (83437 to 148960)	348.26 (249.4 to 447.12)
United States Virgin Islands	191 (79 to 312)	284.23 (106.96 to 482.43)	268 (31 to 523)	308.39 (31.1 to 624.64)
Albania	5379 (4649 to 6109)	338.57 (290.05 to 387.09)	8322 (4709 to 11934)	346.65 (193 to 500.3)
Algeria	43981 (41363 to 46598)	384.84 (361.91 to 407.76)	82432 (56087 to 108776)	382.48 (260.2 to 504.77)
Armenia	4815 (4130 to 5500)	340.91 (290.39 to 391.43)	7018 (3990 to 10045)	346.91 (195.17 to 498.66)
Azerbaijan	8366 (7431 to 9302)	332.71 (295.34 to 370.15)	15401 (9418 to 21384)	337.61 (206.93 to 468.34)
Botswana	1232 (887 to 1577)	306.43 (221.1 to 391.78)	2255 (919 to 3592)	310.13 (127.66 to 492.73)
Brazil	320208 (306468 to 333947)	378.74 (362.54 to 394.94)	581102 (395683 to 766521)	401.13 (272.87 to 529.38)
China	3084000 (2956115 to 3211886)	460.41 (441.3 to 479.52)	8513004 (3540943 to 13485065)	749.62 (311.74 to 1187.5)

Colombia	75554 (71951 to 79156)	334.15 (318.14 to 350.16)	139667 (97980 to 181353)	334.08 (233.78 to 434.38)
Costa Rica	6380 (5551 to 7208)	330.44 (287.99 to 372.88)	11692 (6785 to 16599)	332.53 (191.66 to 473.4)
Cuba	20040 (18475 to 21606)	270.76 (249.11 to 292.4)	33123 (20712 to 45534)	302.8 (187.96 to 417.64)
Ecuador	12966 (11735 to 14197)	240.79 (217.8 to 263.78)	21431 (13401 to 29460)	239.55 (149.48 to 329.61)
Egypt	57143 (54125 to 60162)	379.94 (358.34 to 401.54)	115010 (78061 to 151958)	374.5 (254.29 to 494.71)
Equatorial Guinea	513 (296 to 731)	374.83 (217.5 to 532.53)	1119 (312 to 1934)	390.55 (114.74 to 667.37)
Fiji	630 (394 to 867)	330.99 (198.15 to 466.96)	1030 (333 to 1734)	340.82 (108.34 to 576.08)
Gabon	1119 (796 to 1442)	367.73 (262.26 to 473.38)	1969 (802 to 3137)	372.94 (153.56 to 592.58)
Georgia	6720 (5924 to 7515)	339.82 (297.73 to 381.91)	8331 (5107 to 11555)	343.74 (208.56 to 478.93)
Grenada	185 (64 to 315)	291.62 (90.6 to 514.21)	224 (4 to 481)	314.11 (5.5 to 678.62)
Indonesia	207837 (200979 to 214695)	339.4 (328.03 to 350.77)	359693 (266957 to 452428)	337.31 (250.45 to 424.17)
Iran (Islamic Republic of)	92590 (88511 to 96670)	399.4 (381.94 to 416.85)	172969 (123509 to 222430)	396.4 (283.06 to 509.73)
Iraq	31053 (28917 to 33190)	383.24 (357.52 to 408.97)	74435 (49216 to 99653)	383.19 (253.53 to 512.84)
Jamaica	3336 (2763 to 3909)	311.33 (258.54 to 364.11)	4848 (2612 to 7083)	311.88 (167.33 to 456.44)
Mauritius	1902 (1477 to 2328)	334.52 (259.2 to 409.84)	3342 (1594 to 5091)	342.36 (159.93 to 525.23)
Mexico	118564 (113782 to 123346)	290.18 (278.58 to 301.78)	201240 (144027 to 258453)	286.22 (204.72 to 367.72)

Panama	5196 (4456 to 5936)	324.1 (278.05 to 370.15)	9361 (5269 to 13454)	329.03 (183.67 to 474.39)
Paraguay	6447 (5610 to 7285)	345.63 (301.77 to 389.49)	11177 (6539 to 15815)	347.6 (203.45 to 491.75)
Peru	29346 (27345 to 31347)	232.86 (217.03 to 248.7)	52770 (35149 to 70390)	235.96 (156.76 to 315.16)
Republic of Moldova	7098 (6250 to 7945)	335.09 (294.55 to 375.62)	10788 (6468 to 15109)	340.96 (202.2 to 479.71)
Saint Lucia	220 (86 to 359)	289.27 (114.29 to 471.65)	384 (51 to 735)	319.68 (40.18 to 620.41)
Saint Vincent and the Grenadines	148 (44 to 260)	288.69 (86.73 to 508.31)	219 (8 to 452)	307.58 (10.65 to 643.36)
Samoa	129 (32 to 234)	329.76 (83.29 to 607.04)	214 (9 to 442)	353.39 (13.05 to 751.19)
South Africa	45472 (42927 to 48018)	321.1 (303.22 to 338.98)	73245 (50568 to 95922)	318.79 (220.4 to 417.17)
Sri Lanka	27657 (25756 to 29558)	321 (298.47 to 343.54)	50641 (33844 to 67438)	325.91 (216.93 to 434.88)
Thailand	119179 (114009 to 124350)	306.25 (292.98 to 319.52)	207451 (135400 to 279503)	285.56 (185.91 to 385.21)
Tonga	85 (13 to 168)	333.95 (52.09 to 666.06)	127 (0 to 286)	351.91 (1.2 to 791.43)
Tunisia	16652 (15241 to 18063)	400.86 (366.54 to 435.19)	29412 (18946 to 39878)	401.1 (257.72 to 544.48)
Turkmenistan	3484 (2883 to 4085)	317.42 (264.41 to 370.43)	6501 (3478 to 9523)	316.14 (169.54 to 462.73)
Uruguay	7040 (6215 to 7864)	320.26 (280.45 to 360.07)	8777 (5260 to 12294)	320.85 (189.82 to 451.87)
Uzbekistan	16965 (15572 to 18359)	321.05 (294.48 to 347.62)	31100 (20478 to 41723)	326.55 (215.86 to 437.27)
Suriname	590 (361 to 820)	298.87 (185 to 413.58)	966 (308 to 1631)	302.74 (95.21 to 513.33)

Belize	261 (109 to 416)	289.67 (128.07 to 453.77)	530 (84 to 990)	302.39 (49.03 to 562.37)
Bolivia (Plurinational State of)	6864 (5998 to 7729)	245.68 (214.53 to 276.84)	11410 (6608 to 16212)	250.32 (145.32 to 355.33)
Cabo Verde	366 (192 to 543)	230.48 (120.57 to 342.43)	560 (130 to 1002)	229.86 (53.27 to 410.63)
Cameroon	6869 (5994 to 7743)	224.28 (194.62 to 253.95)	12516 (7303 to 17729)	224.56 (132.32 to 316.8)
Congo	2218 (1746 to 2689)	358.26 (276.14 to 440.39)	4012 (1938 to 6087)	356.51 (172.5 to 540.52)
Democratic People's Republic of Korea	33338 (31265 to 35411)	323.22 (302.67 to 343.77)	46773 (31579 to 61968)	320.24 (216.02 to 424.46)
Dominican Republic	9719 (8673 to 10766)	295.2 (263.95 to 326.45)	16278 (9638 to 22918)	317.06 (187.64 to 446.47)
El Salvador	7069 (6218 to 7920)	336.41 (295.8 to 377.02)	10173 (6084 to 14262)	341.28 (203.59 to 478.98)
Eswatini	446 (255 to 640)	300.48 (163.99 to 446.11)	706 (181 to 1242)	308.81 (81.93 to 540.75)
Ghana	9273 (8248 to 10298)	223.57 (198.09 to 249.05)	16339 (9917 to 22760)	225.39 (137.82 to 312.96)
Guatemala	12979 (11728 to 14229)	338.25 (304.96 to 371.54)	22405 (13780 to 31031)	339.99 (209.58 to 470.39)
Guyana	531 (312 to 752)	288.19 (171.61 to 405.38)	926 (283 to 1577)	297.99 (90.32 to 508.03)
Honduras	6814 (5935 to 7692)	335.45 (292.73 to 378.16)	11432 (6539 to 16326)	339.68 (195.1 to 484.26)
India	768191 (744621 to 791761)	240.19 (232.71 to 247.67)	1768394 (966535 to 2570252)	343.27 (187.92 to 498.63)
Kenya	18108 (16610 to 19606)	313.4 (287.11 to 339.69)	33476 (21676 to 45276)	313.99 (204.73 to 423.26)
Kiribati	55 (4 to 122)	358.39 (25.89 to 828.89)	94 (0 to 230)	364.98 (0 to 902.82)

Kyrgyzstan	4598 (3904 to 5292)	340.74 (291.52 to 389.97)	8611 (4838 to 12383)	351.19 (197.97 to 504.42)
Lao People's Democratic Republic	4044 (3391 to 4697)	334.25 (279.67 to 388.82)	7764 (4247 to 11281)	342.78 (188.74 to 496.83)
Lesotho	968 (679 to 1259)	320.75 (218.67 to 424.49)	1220 (468 to 1976)	327.42 (125.31 to 531.35)
Maldives	362 (176 to 550)	341.86 (172.62 to 511.63)	861 (171 to 1561)	365.58 (76.93 to 657.32)
Marshall Islands	25 (0 to 67)	295.91 (0 to 904.03)	76 (0 to 251)	468.44 (0 to 1508.93)
Mauritania	1412 (1041 to 1783)	231.53 (170.58 to 292.49)	2541 (1113 to 3970)	233.4 (103.58 to 363.23)
Micronesia (Federated States of)	59 (3 to 129)	355.45 (19.16 to 804.66)	112 (0 to 261)	363.44 (0 to 891.8)
Mongolia	2031 (1587 to 2476)	347.89 (269.34 to 427.57)	4202 (2013 to 6392)	362.41 (173.78 to 551.26)
Morocco	38908 (36578 to 41237)	380.55 (357.39 to 403.72)	66144 (45272 to 87016)	376.21 (257.34 to 495.08)
Myanmar	45867 (43305 to 48428)	338.1 (319.25 to 356.95)	78017 (54245 to 101790)	337.2 (234.56 to 439.84)
Namibia	1394 (1030 to 1758)	308.97 (229.04 to 388.9)	2361 (1000 to 3724)	314.18 (135.29 to 493.2)
Nicaragua	5886 (5072 to 6701)	339.24 (293.63 to 384.84)	11180 (6295 to 16065)	345.5 (194.54 to 496.46)
Nigeria	50599 (47798 to 53400)	204.42 (193.29 to 215.55)	98824 (67347 to 130301)	205.18 (140.26 to 270.09)
Palestine	2998 (2424 to 3572)	389.6 (317.82 to 461.37)	6129 (3067 to 9191)	384.48 (194.11 to 574.86)
Philippines	68334 (65075 to 71592)	339.5 (322.37 to 356.64)	114504 (81200 to 147809)	337.28 (239.41 to 435.14)

Sao Tome and Principe	62 (4 to 135)	226.13 (16.15 to 487.31)	101 (0 to 240)	224.51 (0 to 530.09)
Sudan	20734 (19109 to 22359)	376.39 (347.4 to 405.38)	39630 (26005 to 53255)	374.96 (246.95 to 502.97)
Syrian Arab Republic	15929 (14495 to 17362)	389.89 (354.86 to 424.93)	30934 (19555 to 42313)	386.97 (244.03 to 529.9)
Tajikistan	4689 (3974 to 5405)	315.5 (269.47 to 361.53)	9414 (5251 to 13577)	318.96 (178.88 to 459.03)
Timor-Leste	842 (568 to 1118)	340.67 (219.74 to 462.84)	1387 (487 to 2291)	347.28 (122.11 to 573.19)
Venezuela (Bolivarian Republic of)	36586 (34317 to 38856)	369.05 (346.61 to 391.49)	65249 (43992 to 86506)	355.68 (239.54 to 471.82)
Viet Nam	98433 (94269 to 102598)	328.47 (314.84 to 342.1)	170398 (122585 to 218212)	326.55 (234.92 to 418.18)
Zambia	5312 (4553 to 6072)	306.67 (261.62 to 351.72)	9865 (5507 to 14223)	306.84 (173.48 to 440.2)
Afghanistan	10526 (9427 to 11626)	387.74 (344.87 to 430.61)	17407 (10718 to 24096)	383.65 (237.96 to 529.34)
Angola	10117 (9042 to 11193)	370.01 (327.57 to 412.46)	20854 (12647 to 29060)	372.52 (227.17 to 517.88)
Bangladesh	95510 (91330 to 99691)	237.13 (226.44 to 247.83)	166038 (117863 to 214213)	236.16 (167.67 to 304.66)
Benin	2979 (2423 to 3535)	225.14 (182.88 to 267.39)	5257 (2693 to 7822)	225.16 (117.18 to 333.15)
Bhutan	459 (254 to 665)	232.17 (130.22 to 334.85)	886 (225 to 1555)	239.09 (63.82 to 415.94)
Burkina Faso	5625 (4849 to 6401)	238.16 (204.15 to 272.18)	9645 (5523 to 13767)	240.45 (138.99 to 341.9)
Burundi	3018 (2467 to 3568)	301.83 (241.32 to 362.34)	5642 (2978 to 8307)	299.91 (158.59 to 441.23)
Cambodia	11074 (9952 to 12196)	334.98 (299.81 to 370.15)	20063 (12302 to 27823)	337.27 (207.4 to 467.14)

Central African Republic	1716 (1325 to 2109)	383.29 (279.49 to 502.75)	2761 (1284 to 4240)	381.93 (175.17 to 589.7)
Chad	3298 (2711 to 3885)	226.8 (185.37 to 268.22)	5437 (2858 to 8016)	229.23 (122.02 to 336.43)
Comoros	403 (216 to 591)	304.87 (161.32 to 452.59)	693 (171 to 1225)	312.94 (78.48 to 550.58)
Coted'Ivoire	5952 (5135 to 6768)	227.54 (195.19 to 259.88)	11771 (6734 to 16807)	226.5 (130.77 to 322.22)
Democratic Republic of the Congo	32575 (30482 to 34668)	375.23 (350.15 to 400.3)	59212 (40044 to 78381)	367.92 (249.94 to 485.9)
Djibouti	456 (250 to 664)	313.91 (165.14 to 468.62)	994 (254 to 1742)	321.7 (83.02 to 562.18)
Eritrea	1596 (1216 to 1978)	314.08 (226.95 to 403.92)	3055 (1402 to 4708)	310.14 (142.58 to 477.69)
Ethiopia	36565 (34273 to 38856)	307.1 (287.56 to 326.64)	72566 (49201 to 95931)	307.17 (209.23 to 405.11)
Gambia	608 (373 to 844)	232.92 (140.62 to 326.39)	935 (278 to 1596)	235.39 (73.11 to 398.11)
Guinea	3356 (2772 to 3941)	232.96 (192.4 to 273.52)	4798 (2578 to 7019)	234.11 (127.37 to 340.85)
Guinea-Bissau	378 (195 to 563)	232.68 (113.23 to 360.54)	687 (161 to 1222)	238.91 (56.83 to 425.16)
Haiti	5109 (4378 to 5841)	283.87 (240.17 to 327.58)	8612 (4856 to 12369)	285.65 (161.57 to 409.74)
Liberia	1184 (841 to 1527)	224.57 (159.97 to 289.18)	2140 (894 to 3385)	225.68 (95.95 to 355.41)
Madagascar	7578 (6653 to 8503)	296 (257.96 to 334.05)	14471 (8574 to 20368)	295.77 (176.21 to 415.34)
Malawi	7114 (6229 to 7998)	313.57 (274.65 to 352.5)	11455 (6643 to 16268)	314.99 (184.93 to 445.05)
Mali	5404 (4638 to 6170)	234.08 (198.56 to 269.6)	9824 (5509 to 14138)	235.37 (132.92 to 337.82)

Mozambique	8489 (7524 to 9454)	308.89 (272.65 to 345.13)	14715 (8920 to 20510)	306.53 (187.73 to 425.34)
Nepal	15813 (14439 to 17187)	243.55 (221.84 to 265.25)	26394 (16754 to 36034)	244.71 (155.59 to 333.82)
Niger	4587 (3885 to 5289)	233.51 (195.65 to 271.37)	8828 (4843 to 12814)	233.71 (129.43 to 337.99)
Pakistan	68485 (65197 to 71773)	234.23 (222.47 to 245.99)	117064 (83114 to 151014)	231.18 (164.52 to 297.84)
Papua New Guinea	3021 (2472 to 3569)	338 (263.18 to 412.81)	5689 (2988 to 8391)	345.4 (180.98 to 509.81)
Rwanda	5145 (4409 to 5881)	319.07 (272.16 to 365.98)	10479 (5854 to 15104)	317.76 (178.96 to 456.56)
Senegal	4754 (4044 to 5465)	228.65 (194.38 to 262.92)	8047 (4493 to 11601)	229.39 (129.21 to 329.56)
Sierra Leone	2155 (1694 to 2617)	234.54 (182.25 to 286.83)	3354 (1633 to 5075)	231.62 (114.66 to 348.58)
Solomon Islands	271 (119 to 426)	344.46 (145.65 to 566.6)	479 (78 to 894)	369.82 (58.82 to 697.68)
Somalia	4569 (3878 to 5260)	309.46 (251.03 to 368.62)	7333 (3935 to 10732)	311.81 (167.2 to 456.96)
South Sudan	2831 (2290 to 3373)	295.63 (237.57 to 353.69)	5401 (2827 to 7975)	300.09 (158.23 to 441.95)
Togo	2113 (1655 to 2572)	233.11 (179.86 to 286.35)	4072 (1945 to 6199)	233.99 (113.35 to 354.62)
Uganda	11643 (10485 to 12800)	305.41 (274.48 to 336.34)	21651 (13482 to 29819)	308.6 (194.03 to 423.16)
United Republic of Tanzania	21240 (19593 to 22888)	293.2 (270.52 to 315.88)	36891 (24140 to 49641)	288.06 (189.39 to 386.73)
Vanuatu	136 (36 to 245)	321.9 (78.39 to 615.09)	222 (6 to 466)	352.21 (8.94 to 743.35)
Yemen	14412 (13093 to 15731)	397.91 (360.53 to 435.28)	24939 (15724 to 34154)	397.91 (252.19 to 543.63)
Zimbabwe	5323 (4578 to 6068)	305.85 (260.58 to 351.13)	8308 (4744 to 11871)	308.38 (177.65 to 439.11)

Abbreviations: ASIR: age-standardized incidence rate; UI: uncertainty intervals.

Figure S1. ASIR and net drift of AD and other dementias (1992–2021). Panel A. ASIR (2021). Panel B. Net drift (1992–2021). AD – Alzheimer disease, ASIR – age-standardised incidence rate.

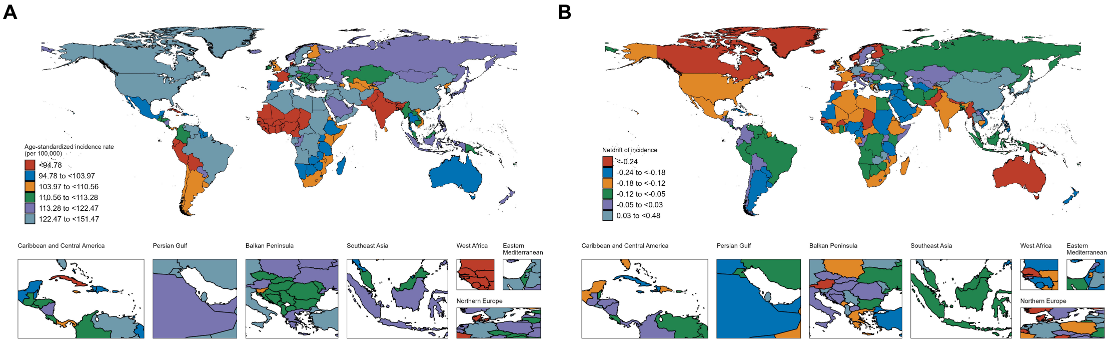

Figure S2. Temporal changes and local drift in incidence rates of AD and other dementias across age groups (1992–2021).* Panel A. Temporal changes. Panel B. Local drift. AD – Alzheimer disease. *The shaded areas represent the annual percentage change in incidence rates (% per year) and the corresponding 95% confidence intervals.

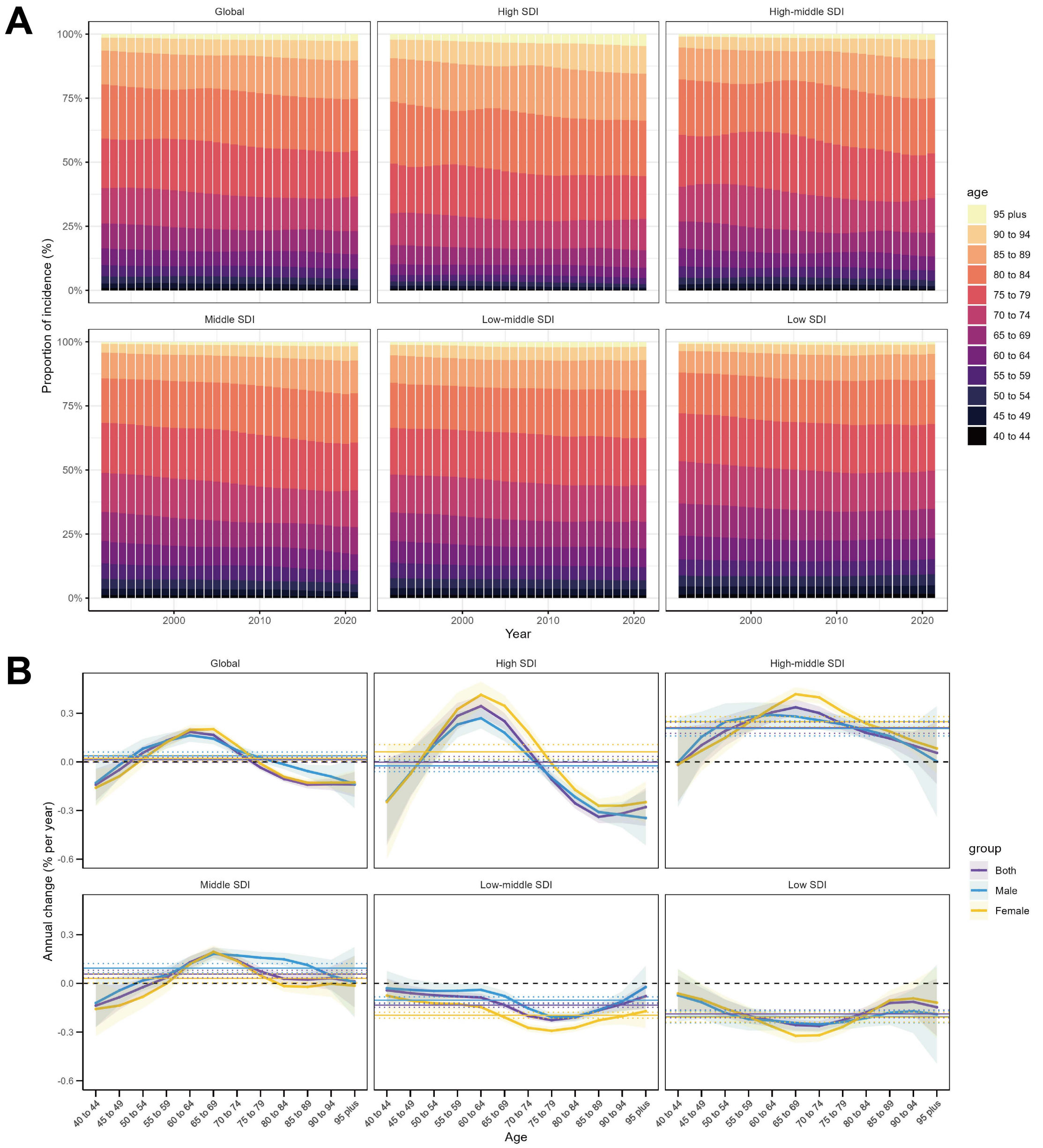

Figure S3. Age, period, and birth cohort effects of typical countries.

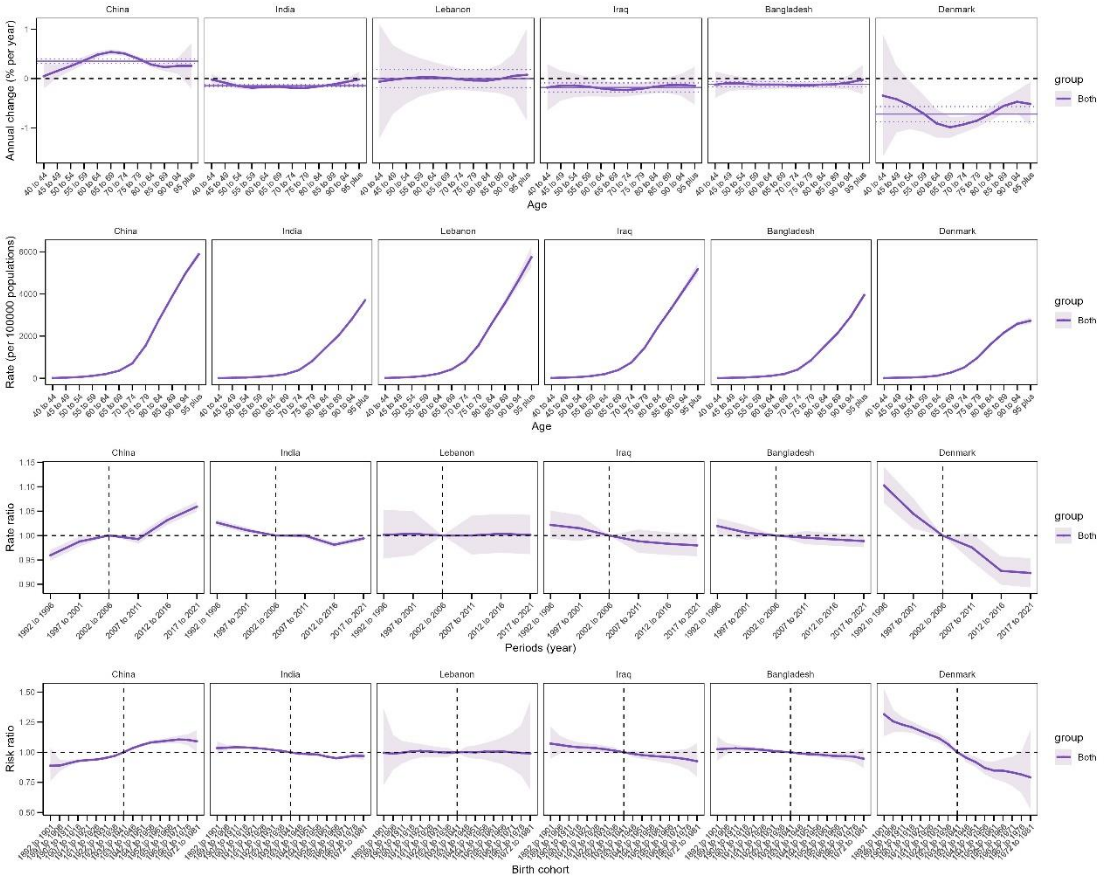

Supplement: Online Supplementary Document [file jogh-15-04156-s001.pdf]
